# Identification of Recessively Inherited Genetic Variants Potentially Linked to Pancreatic Cancer Risk

**DOI:** 10.3389/fonc.2021.771312

**Published:** 2021-12-03

**Authors:** Ye Lu, Manuel Gentiluomo, Angelica Macauda, Domenica Gioffreda, Maria Gazouli, Maria C. Petrone, Dezső Kelemen, Laura Ginocchi, Luca Morelli, Konstantinos Papiris, William Greenhalf, Jakob R. Izbicki, Vytautas Kiudelis, Beatrice Mohelníková-Duchoňová, Bas Bueno-de-Mesquita, Pavel Vodicka, Hermann Brenner, Markus K. Diener, Raffaele Pezzilli, Audrius Ivanauskas, Roberto Salvia, Andrea Szentesi, Mateus Nóbrega Aoki, Balázs C. Németh, Cosimo Sperti, Krzysztof Jamroziak, Roger Chammas, Martin Oliverius, Livia Archibugi, Stefano Ermini, János Novák, Juozas Kupcinskas, Ondřej Strouhal, Pavel Souček, Giulia M. Cavestro, Anna C. Milanetto, Giuseppe Vanella, John P. Neoptolemos, George E. Theodoropoulos, Hanneke W. M. van Laarhoven, Andrea Mambrini, Stefania Moz, Zdenek Kala, Martin Loveček, Daniela Basso, Faik G. Uzunoglu, Thilo Hackert, Sabrina G. G. Testoni, Viktor Hlaváč, Angelo Andriulli, Maurizio Lucchesi, Francesca Tavano, Silvia Carrara, Péter Hegyi, Paolo G. Arcidiacono, Olivier R. Busch, Rita T. Lawlor, Marta Puzzono, Ugo Boggi, Feng Guo, Ewa Małecka-Panas, Gabriele Capurso, Stefano Landi, Renata Talar-Wojnarowska, Oliver Strobel, Xin Gao, Yogesh Vashist, Daniele Campa, Federico Canzian

**Affiliations:** ^1^ Genomic Epidemiology Group, German Cancer Research Center (DKFZ), Heidelberg, Germany; ^2^ Medical Faculty Heidelberg, University of Heidelberg, Heidelberg, Germany; ^3^ Department of Biology, University of Pisa, Pisa, Italy; ^4^ Division of Gastroenterology and Research Laboratory, Fondazione Istituto di Ricovero e Cura a Carattere Scientifico (IRCCS) Casa Sollievo della Sofferenza, San Giovanni Rotondo, Italy; ^5^ Department of Basic Medical Sciences, Laboratory of Biology, Medical School, National and Kapodistrian University of Athens, Athens, Greece; ^6^ Pancreato-Biliary Endoscopy and Endosonography Division, Pancreas Translational and Clinical Research Center, San Raffaele Scientific Institute Istituto di Ricovero e Cura a Carattere Scientifico (IRCCS), Milan, Italy; ^7^ Department of Surgery, Medical School, University of Pécs, Pécs, Hungary; ^8^ Oncological Department, Oncological Unit of Massa Carrara, Azienda Unità Sanitaria Locale (USL) Toscana Nord Ovest, Carrara, Italy; ^9^ General Surgery, Department of Translational Research and New Technologies in Medicine and Surgery, University of Pisa, Pisa, Italy; ^10^ Endoscopic Surgery Department, Hippocratio General Hospital of Athens, Athens, Greece; ^11^ Department of Molecular and Clinical Cancer Medicine, University of Liverpool, Liverpool, United Kingdom; ^12^ Department of General, Visceral and Thoracic Surgery, University Medical Center Hamburg-Eppendorf, Hamburg, Germany; ^13^ Department of Gastroenterology, Institute for Digestive Research, Medical Academy, Lithuanian University of Health Sciences, Kaunas, Lithuania; ^14^ Department of Oncology, Faculty of Medicine and Dentistry, Palacky University Olomouc and University Hospital Olomouc, Olomouc, Czechia; ^15^ Department for Determinants of Chronic Diseases (DCD), National Institute for Public Health and the Environment (RIVM), Bilthoven, Netherlands; ^16^ Department of Molecular Biology of Cancer, Institute of Experimental Medicine of the Czech Academy of Sciences, Prague, Czechia; ^17^ Faculty of Medicine and Biomedical Center in Pilsen, Charles University, Pilsen, Czechia; ^18^ First Faculty of Medicine, Institute of Biology and Medical Genetics, Prague, Czechia; ^19^ Division of Clinical Epidemiology and Aging Research, German Cancer Research Center (DKFZ), Heidelberg, Germany; ^20^ Division of Preventive Oncology, German Cancer Research Center (DKFZ) and National Center for Tumor Diseases (NCT), Heidelberg, Germany; ^21^ German Cancer Consortium (DKTK), German Cancer Research Center (DKFZ), Heidelberg, Germany; ^22^ Department of General Surgery, University of Heidelberg, Heidelberg, Germany; ^23^ Department of Internal Medicine, San Carlo Hospital, Potenza, Italy; ^24^ Department of General and Pancreatic Surgery, The Pancreas Institute, University and Hospital Trust of Verona, Verona, Italy; ^25^ Institute for Translational Medicine, Medical School, University of Pécs, Pécs, Hungary; ^26^ Centre for Translational Medicine, Department of Medicine, University of Szeged, Szeged, Hungary; ^27^ Laboratory for Applied Science and Technology in Health, Carlos Chagas Institute, Curitiba, Brazil; ^28^ First Department of Medicine, University of Szeged, Szeged, Hungary; ^29^ Department of Surgery-Dipartimento di Scienze Chirurgiche Oncologiche e Gastroenterologiche (DiSCOG), Padua University Hospital, Padua, Italy; ^30^ Department of Hematology, Transplantation and Internal Medicine, Medical University of Warsaw, Warsaw, Poland; ^31^ Department of Radiology and Oncology, Institute of Cancer of São Paulo (ICESP), São Paulo, Brazil; ^32^ Faculty of Medicine, University of São Paulo, São Paulo, Brazil; ^33^ Department of Surgery, Faculty Hospital Kralovske Vinohrady and Third Faculty of Medicine, Charles University, Prague, Czechia; ^34^ Digestive and Liver Disease Unit, Sant’ Andrea Hospital, Rome, Italy; ^35^ Faculty of Medicine and Psychology, Sapienza University of Rome, Rome, Italy; ^36^ Blood Transfusion Service, Children’s Hospital, Azienda Ospedaliero-Universitaria Meyer, Florence, Italy; ^37^ Pándy Kálmán Hospital of Békés County, Gyula, Hungary; ^38^ Institute of Molecular and Translational Medicine, Faculty of Medicine and Dentistry, Palacky University Olomouc, Olomouc, Czechia; ^39^ Biomedical Center, Faculty of Medicine in Pilsen, Charles University, Pilsen, Czechia; ^40^ Division of Experimental Oncology, Gastroenterology and Gastrointestinal Endoscopy Unit, Vita-Salute San Raffaele University, Istituto di Ricovero e Cura a Carattere Scientifico (IRCCS) San Raffaele Scientific Institute, Milan, Italy; ^41^ First Propaedeutic University Surgery Clinic, Hippocratio General Hospital, Medical School, National and Kapodistrian University of Athens, Athens, Greece; ^42^ Department of Medical Oncology, Cancer Center Amsterdam, Amsterdam University Medical Centers, University of Amsterdam, Amsterdam, Netherlands; ^43^ Department of Medicine (DIMED), Padua University Hospital, Padua, Italy; ^44^ Department of Surgery, University Hospital Brno Bohunice, Faculty of Medicine, Masaryk University, Brno, Czechia; ^45^ Department of Surgery I, Faculty of Medicine and Dentistry, Palacky University Olomouc and University Hospital Olomouc, Olomouc, Czechia; ^46^ Division of Gastroenterology and Digestive Endoscopy, Department of Gastroenterology, Humanitas Clinical and Research Center Istituto di Ricovero e Cura a Carattere Scientifico (IRCCS), Milan, Italy; ^47^ Department of Surgery, Cancer Center Amsterdam, Amsterdam University Medical Centers, University of Amsterdam, Amsterdam, Netherlands; ^48^ Applied Research on Cancer (ARC)-Net Research Center, University and Hospital Trust of Verona, Verona, Italy; ^49^ Division of General and Transplant Surgery, Pisa University Hospital, Pisa, Italy; ^50^ Department of Digestive Tract Diseases, Medical University of Lodz, Lodz, Poland; ^51^ Centre for Surgical Oncology, Medias Klinikum Burghausen, Burghausen, Germany

**Keywords:** pancreatic cancer, susceptibility, genome-wide association study, recessive model, genetic polymorphisms

## Abstract

Although 21 pancreatic cancer susceptibility loci have been identified in individuals of European ancestry through genome-wide association studies (GWASs), much of the heritability of pancreatic cancer risk remains unidentified. A recessive genetic model could be a powerful tool for identifying additional risk variants. To discover recessively inherited pancreatic cancer risk loci, we performed a re-analysis of the largest pancreatic cancer GWAS, the Pancreatic Cancer Cohort Consortium (PanScan) and the Pancreatic Cancer Case-Control Consortium (PanC4), including 8,769 cases and 7,055 controls of European ancestry. Six single nucleotide polymorphisms (SNPs) showed associations with pancreatic cancer risk according to a recessive model of inheritance. We replicated these variants in 3,212 cases and 3,470 controls collected from the PANcreatic Disease ReseArch (PANDoRA) consortium. The results of the meta-analyses confirmed that rs4626538 (7q32.2), rs7008921 (8p23.2) and rs147904962 (17q21.31) showed specific recessive effects (p<10^−5^) compared with the additive effects (p>10^−3^), although none of the six SNPs reached the conventional threshold for genome-wide significance (p < 5×10^−8^). Additional bioinformatic analysis explored the functional annotations of the SNPs and indicated a possible relationship between rs36018702 and expression of the *BCL2L11* and *BUB1* genes, which are known to be involved in pancreatic biology. Our findings, while not conclusive, indicate the importance of considering non-additive genetic models when performing GWAS analysis. The SNPs associated with pancreatic cancer in this study could be used for further meta-analysis for recessive association of SNPs and pancreatic cancer risk and might be a useful addiction to improve the performance of polygenic risk scores.

## Introduction

Pancreatic cancer ranks fourth for cancer-related deaths in western countries and is projected to become the second by 2030 (1, 2). It is a very deadly disease with the mortality rate closely approaching to the incidence rate. The median survival is less than 18 months, and the 5-year survival rate remains as low as 3 ~ 15% (3–5). The poor prognosis is mainly due to the late onset of symptoms, diagnosis at an advanced stage and subsequent rapid progression. A comprehensive identification of the risk factors can be instrumental to a better understanding of the disease etiology and to the development of methods for risk stratification, that in turn could facilitate early detection, which at the moment remains elusive.

Genetic factors play an important role in the etiology of pancreatic cancer (6). Genome-wide association studies (GWAS) have identified various frequent genetic variants associated with pancreatic cancer risk. The two largest pancreatic cancer GWAS done in European populations are the Pancreatic Cancer Cohort Consortium (PanScan) and the Pancreatic Cancer Case-Control Consortium (PanC4), and a total of 21 susceptibility loci associated at genome-wide significance level have been discovered, and studied individually and in combination (7–15). However, the identified SNPs explain only 4.1% of the total phenotypic variance of pancreatic cancer, which do not fully account for the overall 21.2% estimated genetic heritability (16). This can be explained by the relatively small effect sizes of the individual risk loci, and by the strict multiple testing correction required for GWAS (typically p < 5x10^-8^), which is likely to result in a large number of false negatives.

Over the past decade, GWAS have achieved substantial success in discovering many common variants underlying the genetic architecture of complex diseases (17), including pancreatic cancer. Standard models for implying specific relationships between genotypes and phenotypes include additive, recessive and dominant models (18). The association of biallelic single nucleotide polymorphisms (SNPs) having alleles A/a with a given endpoint (e.g. disease risk) is typically analyzed with a logistic regression model logit(P) = α + β (X), where in an additive model X = 0, 1 or 2 depending on the genotype (homozygotes A/A, heterozygotes A/a and homozygotes a/a, respectively), thus the risk of disease is increased exp(β)-fold for subjects with genotype A/a and exp(2β)-fold for subjects with genotype a/a. A recessive model compares rare homozygotes a/a (who will have X=1) versus the rest (combining heterozygotes A/a and common homozygotes A/A, who will have X=0); a dominant model compares A/A (X=0) versus A/a + a/a (X=1). As most GWAS studies assume that allelic effects are additive, most of the associations reported in GWAS consider only the additive model of inheritance. But for variants which do not follow an intermediate model of inheritance, the recessive or the dominant genetic model can have more power to detect associations. Reanalysis of GWAS data with the recessive model of inheritance, considering homozygotes for the minor allele as the only “exposed” category could help to identify additional risk loci for non-negligible subsets of SNPs (19).

To discover novel recessively inherited pancreatic cancer risk loci, we performed a secondary analysis using genotyping data from all published pancreatic cancer GWAS conducted in subjects of European origin, and then replicated the most promising variants in cases and controls collected from the PANcreatic Disease ReseArch (PANDoRA) consortium. Better understanding the genetic background of the disease could be an invaluable tool to stratify the population by individual risk and increase our chances of early detection.

## Materials and Methods

### Study Populations

The following publicly available GWAS datasets on pancreatic cancer risk were used for this study: the Pancreatic Cancer Cohort Consortium (PanScan, comprising of PanScan I, PanScan II, and PanScan III) and the Pancreatic Cancer Case Control Consortium (PanC4). We obtained the genotype data from the NCBI database of genotypes and phenotypes (dbGaP) (study accession numbers phs000206.v5.p3 and phs000648.v1.p1; project reference #12644). We performed standard quality control and genotype imputation for the four datasets separately, using the Michigan Imputation Server (https://imputationserver.sph.umich.edu) (20) and the Haplotype Reference Consortium (HRC, V.r1.1) reference panel (21). Before imputation, we implemented individual- and SNP-level quality control steps as follows: individual and SNP missingness (call rate<0.9); sex discrepancy; heterozygosity (>3 SD from the mean); relatedness (PI_HAT>0.2, i.e., subjects related up to the second degree); ethnic outliers (population structure was captured by principal component analysis to remove non-European ancestry individuals); minor allele frequency (MAF) <0.005; and Hardy-Weinberg equilibrium (HWE) (p<1×10^−6^). After imputation, we removed SNPs with low imputation quality (INFO score r^2^<0.7, MAF<0.05 or call rate<0.9). Then, we merged the four imputed datasets and rechecked for the relatedness in the pooled dataset. At the end, a total of 5,056,279 SNPs in 8,769 cases and 7,055 controls (8,600 males and 7,224 females) remained for further analysis.

Additional samples belonging to the PANDoRA consortium, mostly from European populations, were selected for genotyping. Cases were diagnosed with pancreatic ductal adenocarcinoma (PDAC) and were all collected from the PANDoRA consortium (22). Controls were from the same geographical regions as the cases. A subset of the German controls (N=932) derived from ESTHER, a prospective cohort with 9,953 participants recruited in the Saarland region of Germany during a general health check-up in the period of July 2000 and December 2002. British and Dutch controls were collected from the European Prospective Investigation on Cancer (EPIC, http://epic.iarc.fr/), a prospective cohort study consisting of general population healthy volunteers from ten European countries (23). All subjects provided written informed consent. Approval for the PANDoRA study protocol (including for controls from ESTHER and EPIC cohorts) was received from the Ethics Commission of the Medical Faculty of the University of Heidelberg.

### SNP Selection

We performed the association analysis on the pooled imputed PanScan+PanC4 GWAS data using both additive and recessive models. Association statistics (odds ratios (OR) and 95% confidence intervals (CI)) on PDAC risk were obtained with logistic regression adjusting for age, sex and the top ten principal components using PLINK version 1.9 (24). There were 268 SNPs that showed an association with p-value lower than 10^-5^, according to a recessive model (**Supplementary Table 1**). Most of them overlapped with previously reported pancreatic cancer risk loci (1q32.1, 2p14, 3q28, 5p15.33, 7p14.1, 7q32.3, 9q34, 13q12.2 and 16q23.1) from additive analyses. Among remaining SNPs which were over 1 Mb away from the closest known locus and showed no linkage disequilibrium (LD) with known loci (r^2^ < 0.01), ten SNPs at six loci, showed large differences in p-values using the two models (p < 10^-5^ using the recessive model, and p > 10^-3^ using the additive model). After filtering SNPs in LD (r^2^>0.8, N=3) and removing SNPs that showed p≥0.05 for association with PDAC risk in either PanScan or PanC4 (N=1), the top six promising SNPs were moved forward to genotyping.

### Genotyping

DNA of PANDoRA samples was isolated from whole blood using QIAamp DNA extraction kit (Qiagen) and distributed in 384-well plates for genotyping. For quality control, 8% of the samples was randomly duplicated throughout the plates and no template controls (NTC) were used in each genotyping plate. Genotyping was performed using TaqMan (ABI, Applied Biosystems, Foster City, CA, USA) and KASP (KBioscence, Hoddesdon, UK) probes on the Real-Time PCR system. Since the genotyping assay for rs147904962 failed to work, rs12943205 was genotyped as a proxy SNP, in high LD (r^2^ = 0.99). Detection was done with a Viia7 instrument and Viia7 software (Applied Biosystems, Foster City, CA). After calling all the genotypes, samples with a call rate < 83.3% (i.e., missing more than one genotype) were removed. Duplicated samples with low concordance rate (>1 discordant genotype) were excluded. Discordance from HWE distribution was checked in controls, in the overall population and by country, and all the genotyped SNPs were in HWE (p>10^-3^). Dutch and British controls were genotyped in the context of a GWAS using the Human 660W-Quad BeadChip array (Illumina, San Diego, CA). Quality control steps were performed after TaqMan genotyping. Finally, 3,212 PDAC cases and 3,470 controls were included for further analysis. The characteristics of the study population are summarized in **Table 1**.

**Table 1:** Characteristics of genotyped samples from PANDoRA after quality control.

	Cases	Controls
Male, %	55.0	51.6
Median age, (25th-75th percentile)	66 (58-73)	60 (51-68)
Country, N		
Czech Republic	430	173
Germany	683	1018
Greece	109	16
Hungary	290	413
Italy	1298	1280
Lithuania	102	179
Poland	90	195
Netherlands	117	62
United Kingdom	93	134
Total	3212	3470

### Statistical Analysis

To investigate the effect of the genotyped SNPs (rare allele vs. common allele; rare homozygous genotype vs. heterozygous plus common homozygous genotypes) in PANDoRA samples on the PDAC risk, we performed unconditional logistic regression adjusting for sex, age and country. Then we performed meta-analyses using R package “meta” by fixed-effects model (or random-effects model when p < 0.05 in the heterogeneity test) between phase one (reanalysis of the pancreatic cancer GWASs, PanScan and PanC4) and phase two (replication in samples collected from PANDoRA), with a final sample size of 11,981 PDAC cases and 10,525 controls. For the analysis with the genotyped SNPs in phase two, age, sex and genotypes had missing rates between 1% to 5%. Considering that missing data can have a significant effect on the conclusion, we applied multiple imputation which is a missing data method that provides valid statistical inferences under the missing at random condition (25). The R package “mice”, which imputes incomplete multivariate data by chained equations (26), was used to impute five times the variables involved in analysis, to analyze each of the imputed datasets separately based on the logistic regression model, then to automatically combine all the results together. Since the Brazilian population is known to be ethnically admixed, we performed additional statistical analyses with the PANDoRA Brazilian cases and controls. Meta-analyses were performed after multiple imputation as well. Analyses were carried out with R V3.6.

In addition, we performed gene-based analysis using MAGMA v1.08 to test the associations between all coding genes and PDAC risk based on the p-values under additive and recessive models respectively (27).

### Bioinformatic Tools

We used the following tools/databases to explore the possible function of candidate SNPs: the Genotype-Tissue Expression (GTEx, 8th version) project portal (https://www.gtexportal.org, accessed on 30 June 2020), HaploReg v4.1 (https://pubs.broadinstitute.org/mammals/haploreg/haploreg.php) and RegulomedB (https://www.regulomedb.org/regulome-search/) (28–30). The Gene Expression Profiling Interactive Analysis (GEPIA2) database (http://gepia2.cancer-pku.cn) was applied to verify the expression levels and evaluate the prognostic value of genes of interest in pancreas tumor and normal tissues (31). Three-Dimensional-genome Interaction Viewer (3DIV, http://3div.kr), which collected all publicly available high-throughput chromatin conformation capture (Hi-C) data from human cell/tissue types, was used to explore the locus regulatory effects of the 3D genome (32). SNPnexus (https://www.snp-nexus.org/) and OpenTargets Genetics (https://genetics.opentargets.org) summarize the results of many different functional annotations (33, 34). The Functional Mapping and Annotation of Genome-Wide Association Studies platform (FUMA, https://fuma.ctglab.nl) was used to annotate the results of the recessive model GWAS (35).

## Results

**Figure 1:**
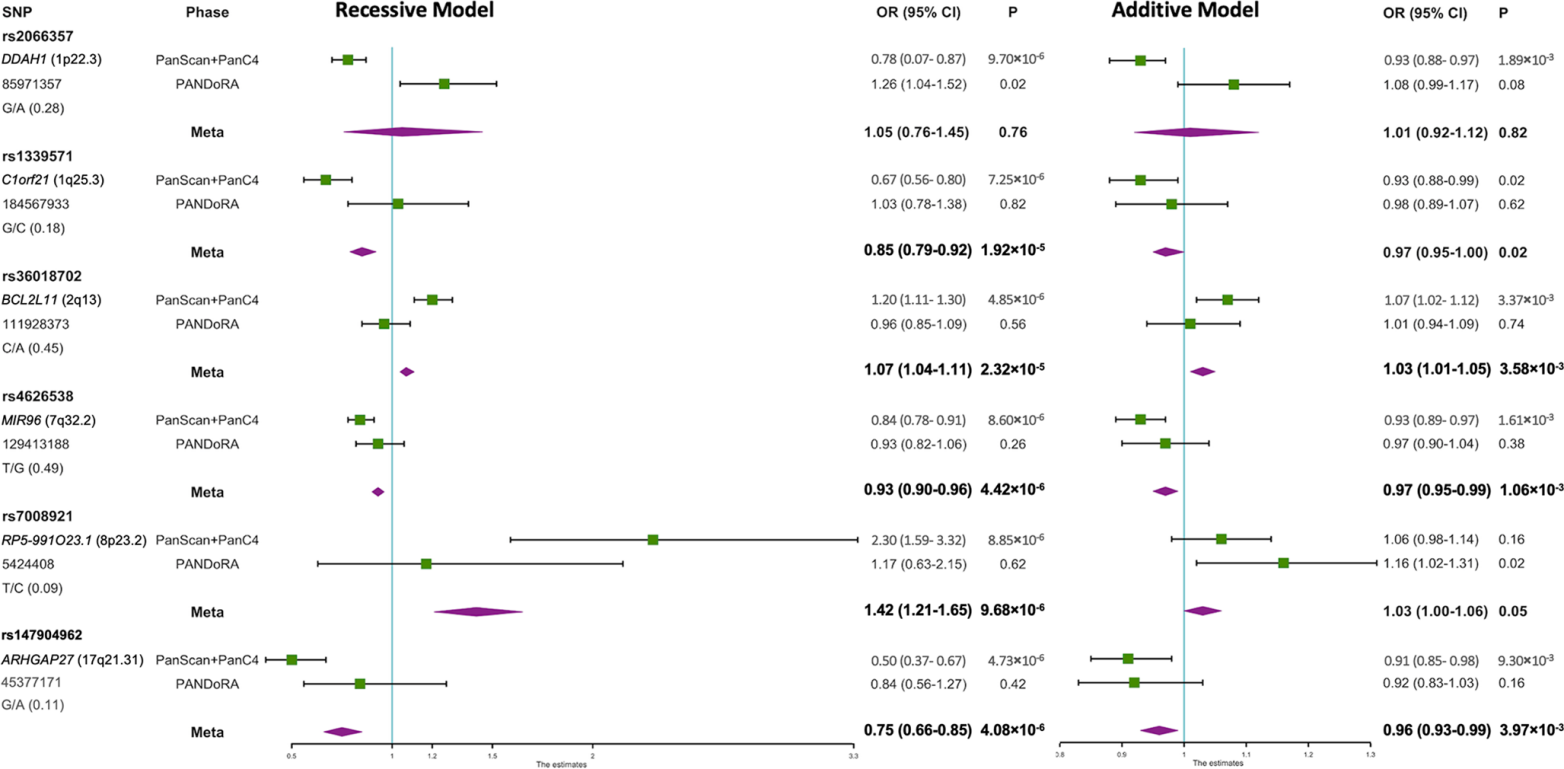
Forest plot of the associations of the 6 SNPs with PDAC risk under recessive and additive genetic models. A forest plot for the 6 SNPs and risk of PDAC is shown by two genetic models using data from discovery and replication analyses combined. Population specific odds ratios (OR) and 95% confidence intervals (CI) are denoted by green boxes and black lines. The combined OR estimates are represented by purple diamonds, where diamond width corresponds to 95% CI bounds. The position information (hg38) and minor allele frequency (MAF) for each SNP are shown on the left.

In the first phase, which was conducted at a genome-wide scale, we re-analyzed the data from the PanScan+PanC4 GWAS dataset according to a recessive model of inheritance, and we observed six SNPs that showed specific recessive associations with PDAC risk with p<10^-5^ while p>10^-3^ using the additive model (**Figure 1**, **Supplementary Table 2**). The correlated SNPs in these regions (r^2^>0.8 in LD) did not show evidence of stronger association under an additive model (**Supplementary Table 3**, **Supplementary Figure 1**). The associations of the genotyped SNPs with PDAC risk under the additive and recessive genetic models are shown in **Figure 1** (**Supplementary Table 2**). In the validation phase in PANDoRA, no statistically significant associations (p<0.05) were observed, using the recessive model, except for rs2066357. However, this SNP showed high heterogeneity, with an opposite effect compared to the discovery phase under the recessive genetic model.

In meta-analyses, none of the six SNPs reached the conventional genome-wide significance threshold (p<5×10^−8^). However, *MIR96* rs4626538 (OR=0.93; p=4.42×10^−6^), *RP5-991O23.1* rs7008921 (OR=1.42; p=9.68×10^−6^) and *ARHGAP27* rs147904962 (OR=0.75; p=4.08×10^−6^) maintained a specific recessive effect compared to the additively inherited effects (p=1.06×10^−3^, p=0.05 and p=3.97×10^−3^, respectively), and the p-values of rs4626538 and rs147904962 in the meta-analysis were slightly lower in comparison with those observed in the first phase. The results after multiple imputation were generally consistent with those without multiple imputation (**Supplementary Table 2**). Results did not change when we added the PANDoRA cases and controls from Brazil, who are ethnically admixed (**Supplementary Tables 4, 5**).

We used data from the GTEx consortium to investigate associations between genetic variants and RNA expression. We observed that the rs147904962-A allele was associated with increased *LRRC37A4P* RNA expression in adipose tissue (p=8.1×10^−6^). An expanded list of linked SNPs (in LD with our six candidate SNPs, r^2^>0.6) was also considered for the GTEx analysis; we found that the T allele of rs590097 (in LD with the A allele of rs36018702, r^2 =^ 0.74, D’=1) was associated with higher expression of *BCL2L11* in pancreas (p=5.64×10^−6^). No expression quantitative trait loci (eQTL) associations in pancreas were found for the other SNPs. Haploreg and RegulomeDB did not show evidence for functional effect for these variants.

Using a threshold of >2 for distance-normalized chromatin interaction frequency, 3DIV predicted *C1orf21* and *APOBEC4* to be interaction genes for rs1339571, *BUB1* for rs36018702, *MIR4423* for rs2066357, *SPPL2C*, *SLC4A1*, *RUNDC3A*, *LOC100133991*, *TEX34*, *ITGA2B*, and *C17orf57* for rs147904962, respectively.

Additional analyses with SNPnexus and OpenTargets Genetics did not suggest any clear functional link between our candidate SNPs and pancreatic physiology or pathology. Likewise, when we reanalyzed with FUMA the results of the GWAS analysis according to the recessive model, we did not observe any noteworthy signal in the regions of the six candidate SNPs.

**Figure 2:**
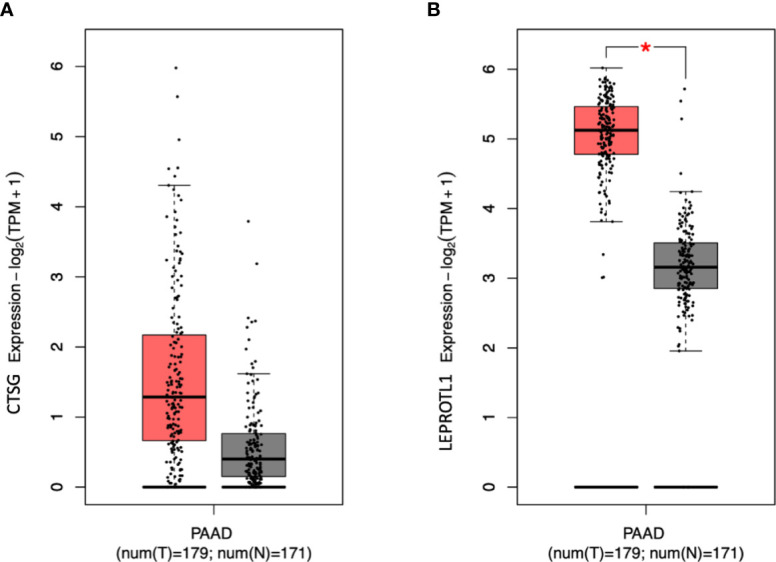
The expression level of *CTSG* and *LEPROTL1* in PAAD patients. GEPIA2 generates box plot for comparing gene expression in pancreatic cancer and paired normal tissues (TCGA tumor versus TCGA normal + GTEx normal). **(A, B)** differential expression analysis. Peach and grey clusters represent tumor and normal samples; * genes with higher |log2FC| values (>1) and lower Q-values (<0.01) were considered differentially expressed genes.

The gene-based analysis using MAGMA based on the p-values of the recessive model revealed that 14 genes were associated with PDAC risk at p < 0.001 (**Supplementary Table 6**). Two of these genes showed evidence for association at p < 0.001 under the recessive model (*CTSG* 14q12, p = 2.53x10^-4^; *LEPROTL1* 8p12, p = 4.34x10^-4^), but not with the additive one (p = 0.20 and p = 0.10, respectively). Then we verified the expression level of the two genes in pancreatic cancer patients using GEPIA2. We found that *LEPROTL1* has increased expression in pancreatic cancer tissues compared to adjacent normal pancreatic tissues of the same patients (**Figure 2**).

## Discussion

GWAS data are usually analyzed according to an additive genetic model, which is generally considered to be a good surrogate for other genetic models, except for the recessive one (19). Researchers have reported risk variants that showed specifically stronger evidence under a recessive model than an additive model, for type 2 diabetes (36), schizophrenia (37), high triglycerides (38), and other traits (39, 40), but not for PDAC yet. To identify recessive susceptibility loci for PDAC risk, we performed a secondary analysis with the largest currently available pancreatic cancer GWAS datasets (PanScan and PanC4) of European ancestry and attempted the replication of the six most promising variants in additional samples collected from the PANDoRA consortium, with a combined sample size of 11,981 PDAC cases and 10,525 controls. In this study, none of our results reached genome-wide statistical significance (p<5x10^−8^) in either phase, or in the meta-analyses, therefore our results are not conclusive. However, for five of the six selected SNPs the results of the meta-analysis do not exclude the possible recessive association with pancreatic cancer risk. In particular, rs4626538 (7q32.2), rs7008921 (8p23.2) and rs147904962 (17q21.31) maintained a large difference in significance between recessive effects compared with the additively inherited effects.

None of the previous studies indicated a link between these loci and pancreatic cancer risk. No variants in high LD (r^2^>0.8) have been previously associated with any trait or disease in GWAS, although variants in low to moderate LD (r^2 =^ 0.14~0.60, D’=0.88~1 in Europeans) with rs147904962 have been reported to be associated with waist-to-hip ratio and with risk of developing allergic diseases. The minor G allele of rs7214661 (r^2 =^ 0.19, D’=0.98) was associated with higher risk of allergic disease (41) while the corresponding A allele of rs147904962 was associated with lower risk of pancreatic cancer in our study. It is consistent with the protective effect of allergy for pancreatic cancer in epidemiologic studies (42).

Additionally, GTEx showed that rs590097 regulates *BCL2L11* expression in pancreas tissue. *BCL2L11* is a member of the BCL2 family and plays a role in neuronal and lymphocyte apoptosis. There is evidence shown that *BCL2L11* is one of the major genes contributing to apoptosis, known to be important for pancreatic biology (http://www.genome.jp/kegg/pathway.html) (43). Moreover, the observed association that rs36018702-A (correlated with rs590097-T) showed increased risk of PDAC is consistent with the higher expression of *BCL2L11* in pancreatic cancer tissues than in normal pancreas tissues found through GEPIA2.

*BUB1* is the interaction gene of rs36018702 predicted by 3DIV. There is evidence that *BUB1* is overexpressed in PDAC tissues, suggesting a role of *BUB1* in PDAC progression, and therefore corroborating the association of rs36018702 and PDAC risk (44).

The lowest p-value we observed in the meta-analysis is 4.08x10^-6^ for the association of rs147904962 (17q21.31) with the risk of PDAC. rs147904962 is situated 17kb at the 3’ end of Rho GTPase Activating Protein 27 (*ARHGAP27*). This gene encodes a member of a large family of proteins that activate Rho-type guanosine triphosphate (GTP) metabolizing enzymes and are involved in cancer through the dysregulation of this mechanism. As *ARHGAP27* mRNA is expressed in pancreatic cancer, we speculate that rs147904962 mediates regulation of cancer-associated *ARHGAP27*, promoting carcinogenesis through dysregulation of Rho/Rac/Cdc42-like GTPases (45). However, it has to be acknowledged that this SNP is not known to be located in a regulatory region of *ARHGAP27*.

Gene-based analyses based on the PanScan and PanC4 datasets (we were not able to replicate these analyses in PANDoRA, which does not have GWAS data) showed that SNPs in *LEPROTL1* and *CTSG* were associated with PDAC risk according to the recessive, but not to the additive model. The bioinformatic analysis identified that *LEPROTL1* was highly expressed in pancreatic cancer compared to matched normal pancreatic tissue of the same patients, suggesting a potential involvement in the etiopathology of PDAC. The leptin receptor overlapping transcript-like 1 gene (*LEPROTL1*) encodes a membrane protein, and may play a role in liver resistance by suppressing the growth hormone activity (46, 47), while the pancreatic cancer-related functions of *LEPROTL1* remain unknown. The cathepsin G gene (*CTSG*) encodes a neutrophil serine protease of the chymotrypsin family, which was shown to affect neutrophil infiltration into the pancreas in a mouse model of pancreatitis (48). Based on this circumstantial evidence it is tempting to speculate a role for this gene and its polymorphisms in modulation of inflammation in the pancreas, which plays a role in the etiology of PDAC. However, to the best of our knowledge, a role for CTSG in pancreatic cancer has not been reported in the literature.

The lack of direct functional evidence for the SNPs of interest from bioinformatic analyses may at least in part reflect the fact that also bioinformatic tools/databases have not been designed to address effects of real recessive alleles. *Ad hoc* tools are needed to better understand the genetic architecture of complex genetic diseases.

It is hard to reach sufficient statistical power to detect variants with recessive effects, unless they are very frequent or have very large effects. Given the effective combined sample size of 11,981 PDAC cases and 10,525 controls, disease prevalence of 1.6%, and a significance cut-off of p<5×10^−8^, we had at least 80% power to detect a association with ORs equal to those observed in the discovery phase for the rare homozygote genotype for SNPs rs7008921 and rs147904962, whereas for the other SNPs power ranged between 54% to 69%. Thus, our study, in spite of the large sample size, lacked statistical power to confirm the risk with recessive model for some of the SNPs. It is worth noting that between PanScan, PanC4 and PANDoRA we have used the largest available resources for genetics of pancreatic cancer in populations of European origin. Our hypothesis that some variants may be associated with pancreatic cancer risk with a recessive model of inheritance was not disproved, but to prove it convincingly will require even larger datasets that will become available as more GWAS on pancreatic cancer risk are performed.

Identifying high-risk groups could contribute to focus surveillance and invasive screening measures, thereby improving the chance of early detection. Polygenic risk scores (PRS) approaches which could combine modest effect from each risk SNPs have demonstrated accuracies between 59% and 63% for predicting the risk of PDAC when including both non-genetic and genetic factors (14, 49–51). The accuracy of the existing PRS is not ready yet to be used in the clinical practice. It is necessary to expand the PRS with additional risk factors to improve its predictive power. For example, PRS including more SNPs that are not genome-wide significant but having noteworthy effects such as the ones we highlighted in this work may provide an additive contribution to the overall performance.

## Conclusions

In conclusion, we propose some candidate SNPs as recessively inherited genetic variants for pancreatic cancer risk in European populations, which should be further confirmed by better powered investigations and/or meta-analysis of our results with those of other studies. Although none of the SNPs reached the genome-wide statistical significance, it is still worth to include these relevant SNPs into the PRS approach for risk stratification. A risk stratification approach with high predictive power could be used to identify subgroups at particularly increased risk of pancreatic cancer, either in the general population or in groups that are already known to have an elevated risk, such as diabetics.

## Data Availability Statement

The datasets presented in this study can be found in online repositories. The names of the repository/repositories and accession number(s) can be found in the article/*Materials and Methods, Study Populations* section.

## Ethics Statement

The studies involving human participants were reviewed and approved by the Ethics Commission of the Medical Faculty of the University of Heidelberg. The patients/participants provided their written informed consent to participate in this study.

## Author Contributions

DC and FC conceived and designed the study. YL performed the lab work. YL performed data curation and analysis. YL drafted the manuscript. DC, MGe, and FC reviewed and edited the manuscript. All other authors provided samples and data. All authors critically read, commented and approved the final manuscript.

## Funding

This work was supported by intramural funding of German Cancer Research Center (DKFZ); and by Fondazione Tizzi (www.fondazionetizzi.it); Fondazione Arpa (www.fondazionearpa.it); the Economic Development and Innovation Operative Programme Grant (Grant number GINOP-2.3.2-15-2016-00048); the Human Resources Development Operational Programme Grant (Grant number EFOP 3.6.2‐16‐2017‐0006); Associazione Italiana Ricerca Cancro (Grant number 5x1000, 12182, and IG 17177); Fondazione Italiana Malattie Pancreas – Ministero Salute (Grant number FIMPCUP_J38D19000690001); Fondazione Cariverona: Oncology Biobank Project “Antonio Schiavi” (Grant number 203885/2017); the Ministry of Health of the Czech Republic (Grant number NV19-03-00097, FNOL-00098892); student grant from Palacky University (Grant number IGA_LF_2020_005). The ESTHER study was funded by the Baden-Württemberg State Ministry of Science, Research and Arts (Stuttgart, Germany).

## Conflict of Interest

The authors declare that the research was conducted in the absence of any commercial or financial relationships that could be construed as a potential conflict of interest.

## Publisher’s Note

All claims expressed in this article are solely those of the authors and do not necessarily represent those of their affiliated organizations, or those of the publisher, the editors and the reviewers. Any product that may be evaluated in this article, or claim that may be made by its manufacturer, is not guaranteed or endorsed by the publisher.
