# Supplementary material for: Identification of Recessively Inherited Genetic Variants Potentially Linked to Pancreatic Cancer Risk
Source: Front Oncol. 2021 Dec 3;11:771312. doi: 10.3389/fonc.2021.771312 (PMC8678088; doi:10.3389/fonc.2021.771312)
Supplement: Supplementary file 1 [file DataSheet_1.docx]

**a)**

**
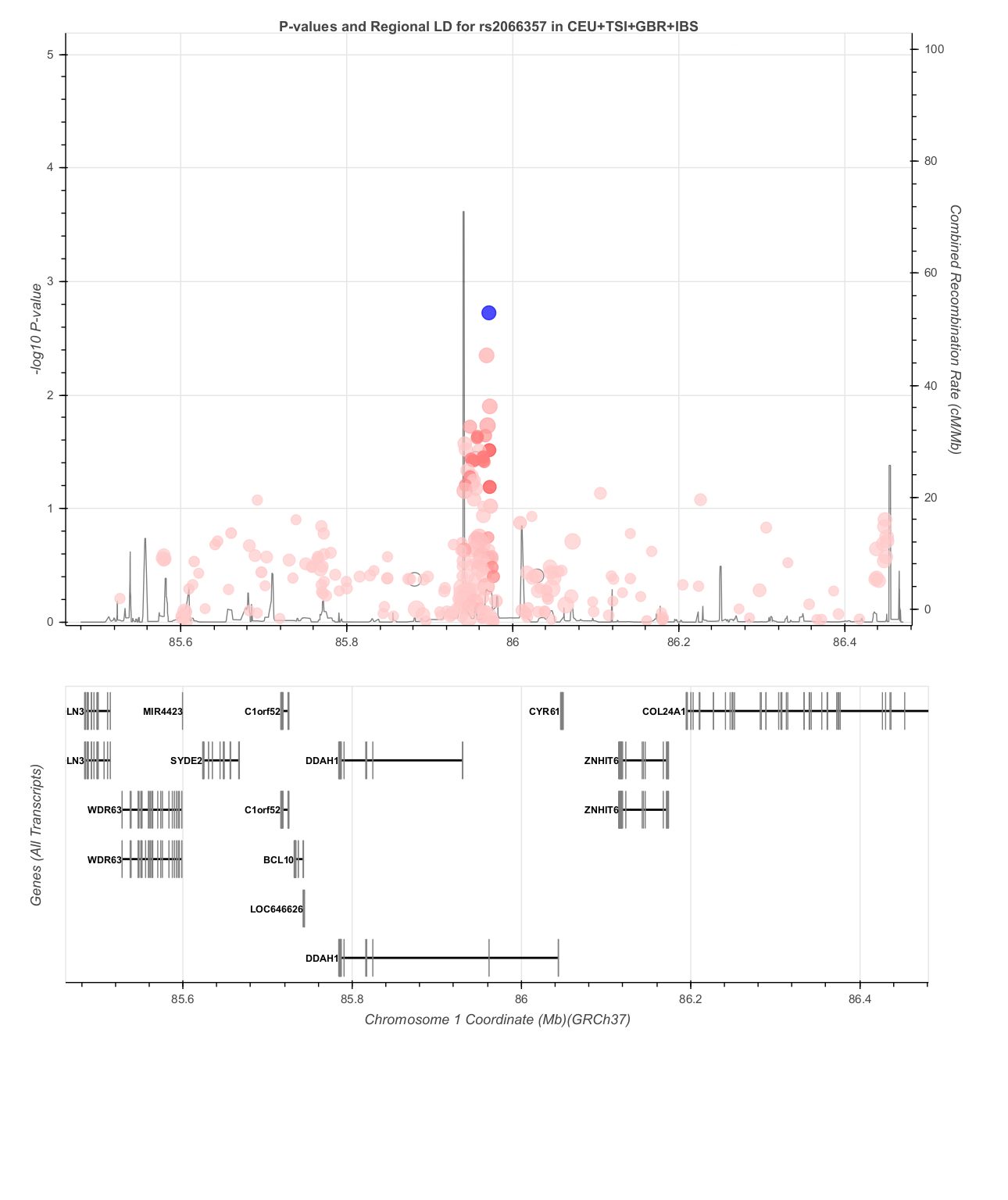
**

**b)**

**
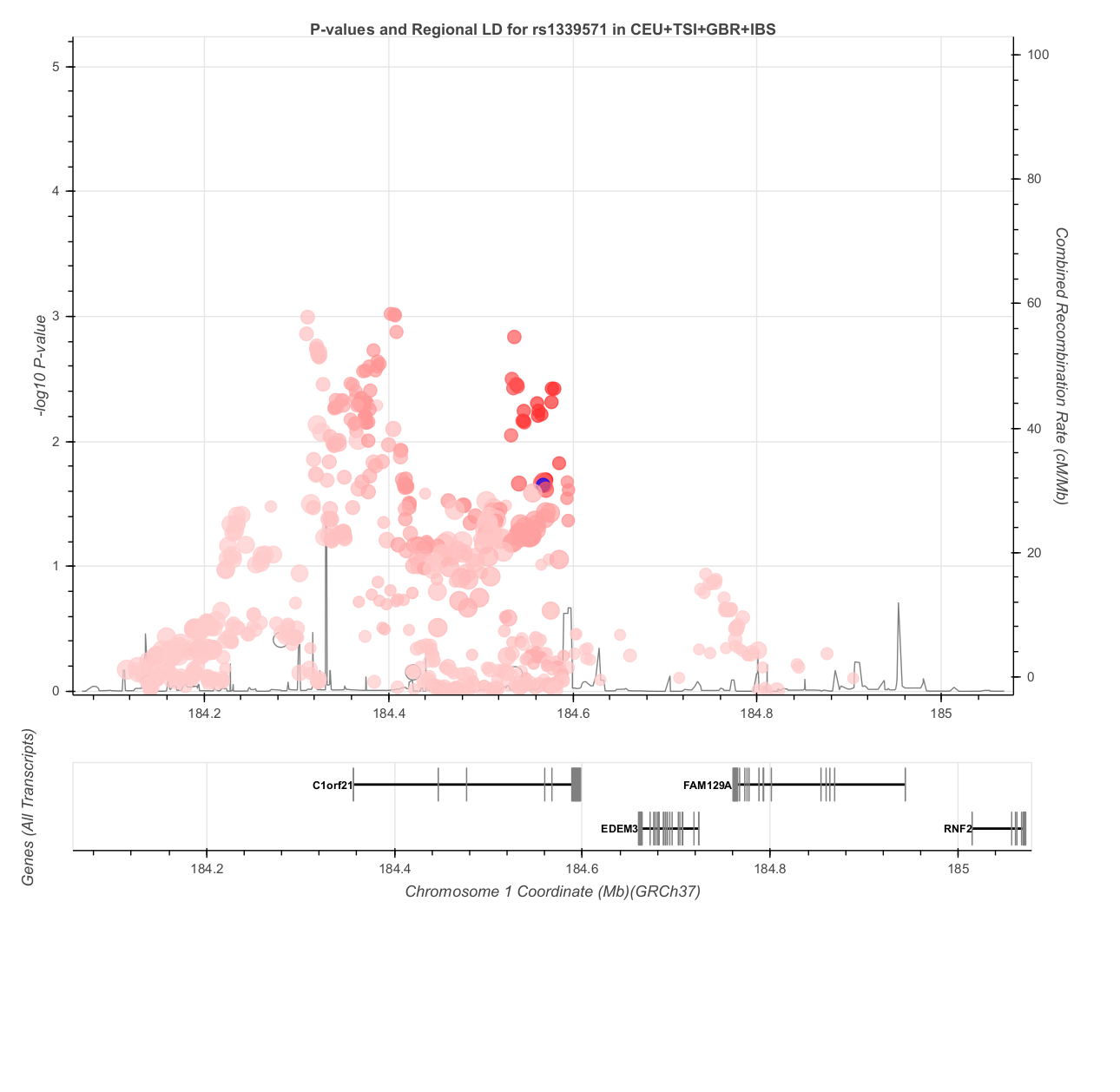
**

**c)
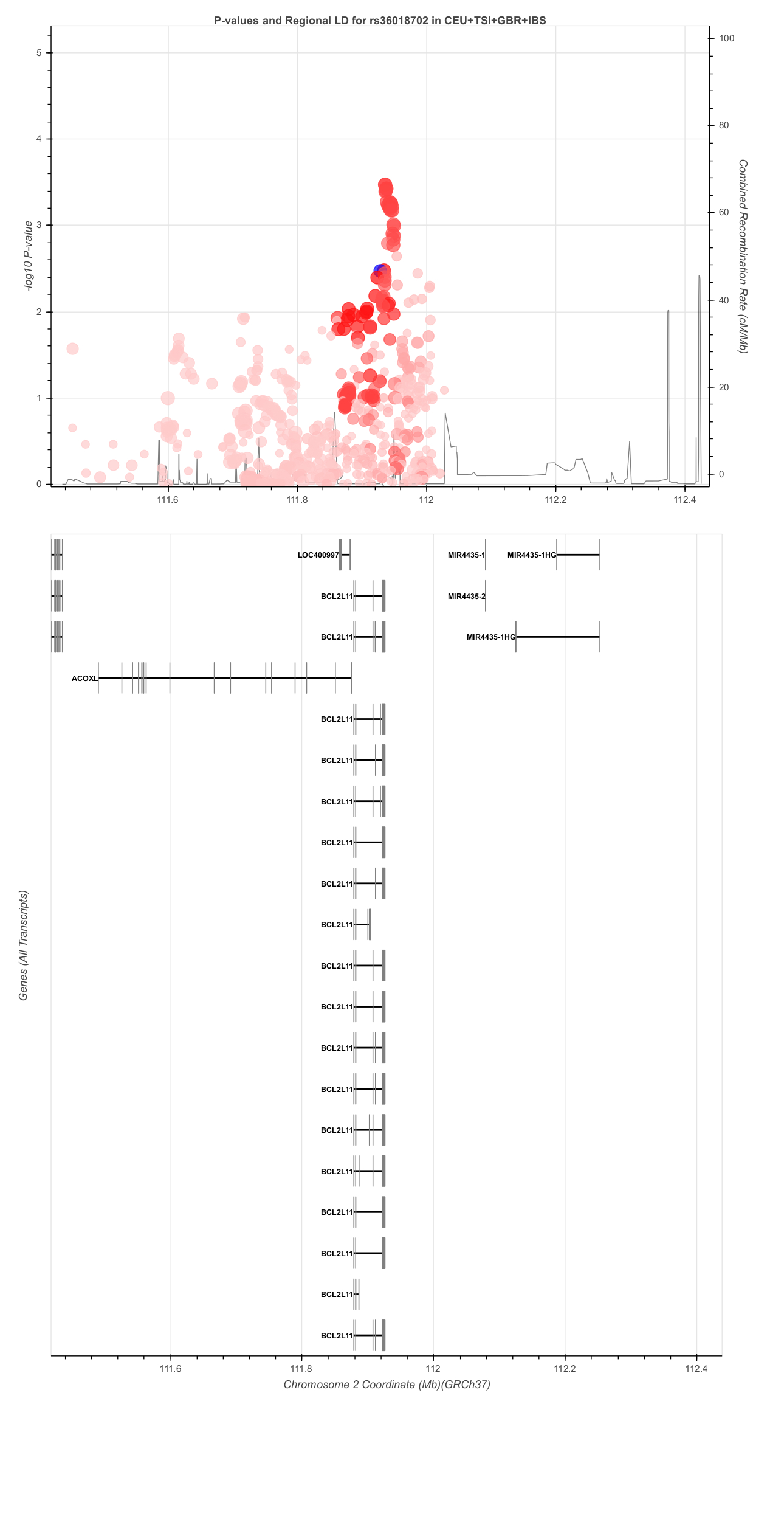
**

**d)**

**
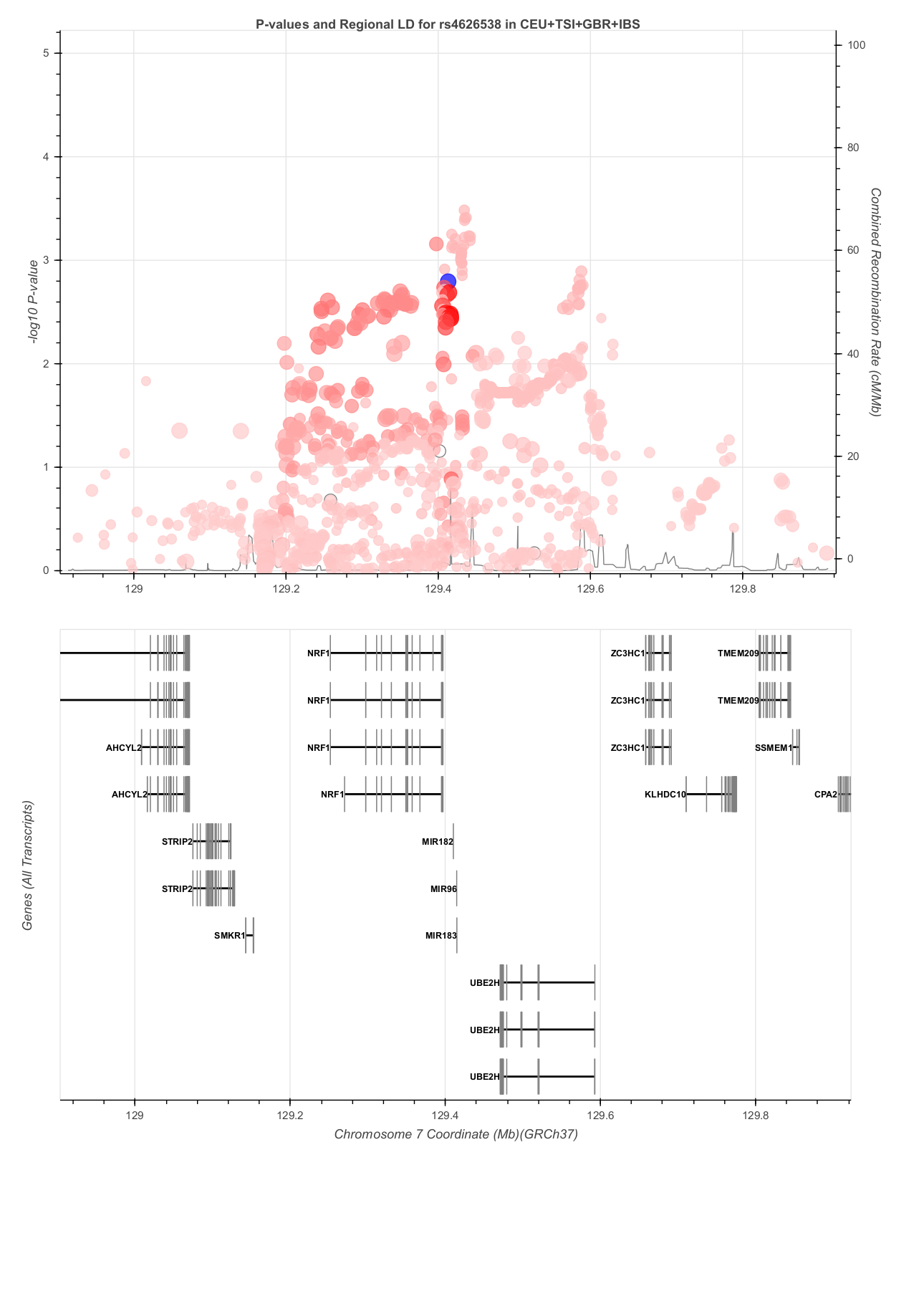
**

**e)**

**
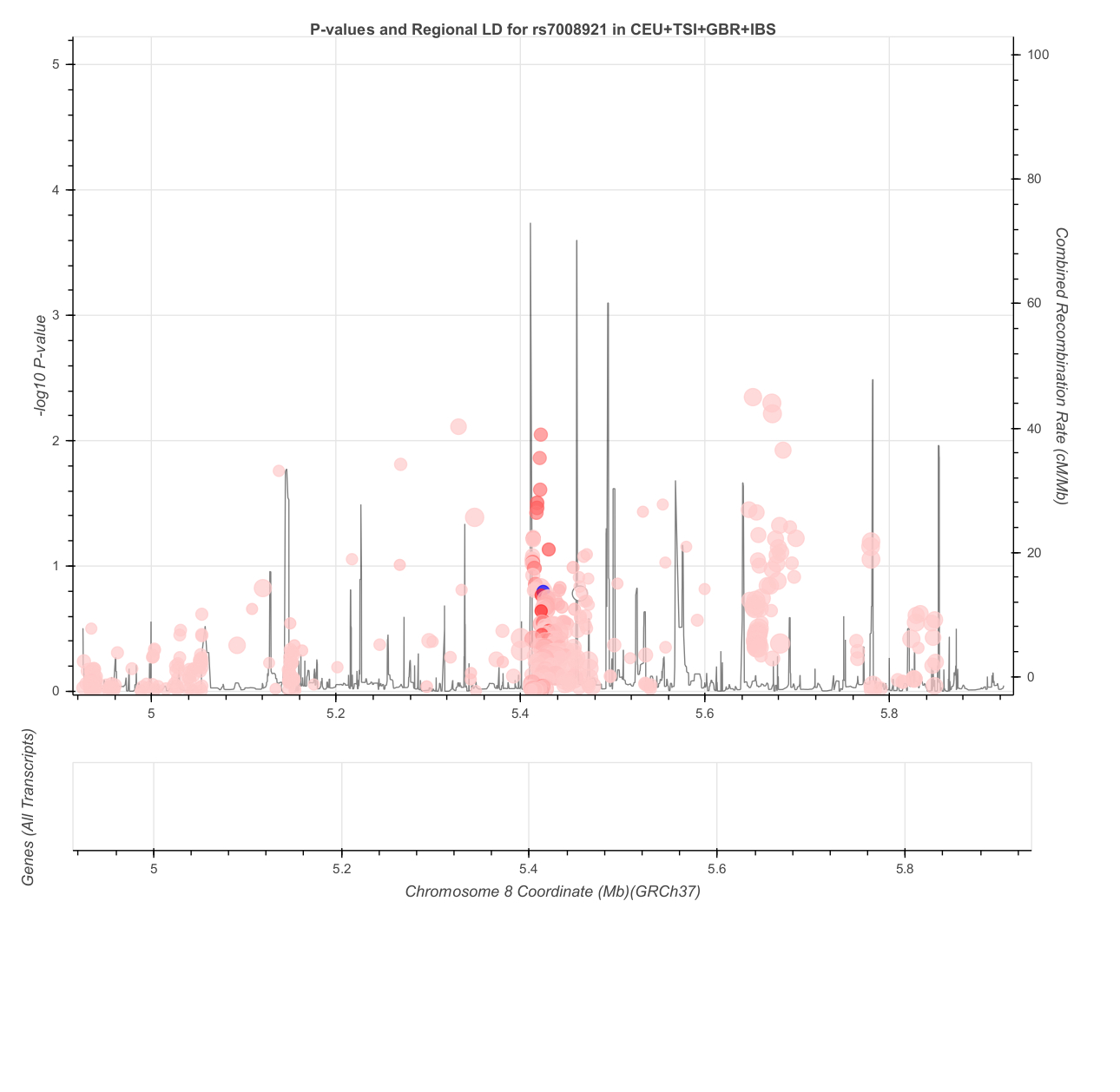
**

**f)**

**
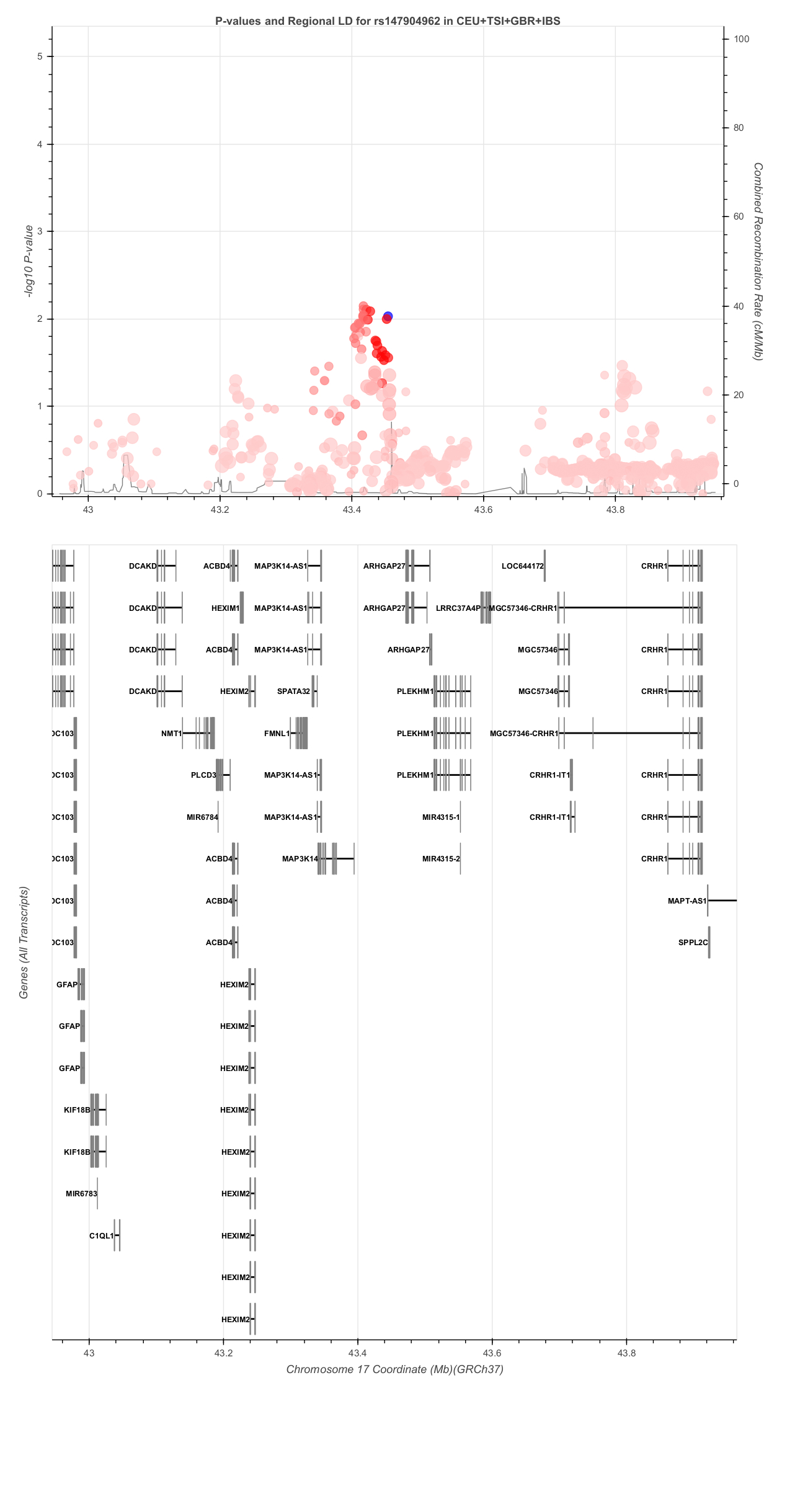
**

**
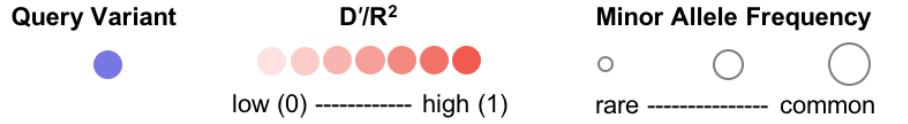
**

**Supplementary Figure 1.** Visualization of association p-value results and linkage disequilibrium patterns for genomic regions of interest. The graphs were generated using the LDassoc web-tool (https://ldlink.nci.nih.gov/?tab=ldassoc), showing the associations obtained from the analyses of PanScan and PanC4 using both additive and recessive models. a)-f) represents for rs2066357, rs1339571, rs36018702, rs4626538, rs7008921, and rs147904962, respectively. In each panel, the left graph generated by p-values under recessive model, and the right graph generated by p-values under additive model

**Supplementary Table 1**. Associations of 268 SNPs showing p<10^-5^, according to the recessive model, and comparison with associations according to the additive model.

| **SNP** | **Chr** | **Position**  **(hg37)** | **Recessive model** | | **Additive model** | |
| --- | --- | --- | --- | --- | --- | --- |
|  |  |  | **OR (95% CI)** | **P-value** | **OR (95% CI)** | **P-value** |
| rs2066357 | 1 | 85,971,357 | 0.78 (0.70-0.87) | 9.70E-06 | 0.93 (0.88-0.97) | 1.89E-03 |
| rs1339571 | 1 | 184,567,933 | 0.67 (0.56-0.80) | 7.25E-06 | 0.93 (0.88-0.99) | 2.24E-02 |
| rs12748835 | 1 | 199,879,616 | 0.81 (0.74-0.89) | 3.87E-06 | 0.86 (0.82-0.90) | 2.55E-10 |
| rs6670899 | 1 | 199,891,438 | 0.80 (0.73-0.87) | 4.93E-07 | 0.86 (0.82-0.90) | 3.61E-11 |
| rs34777525 | 1 | 199,891,555 | 0.80 (0.73-0.88) | 5.93E-06 | 0.85 (0.81-0.89) | 5.23E-11 |
| rs11578938 | 1 | 199,892,279 | 0.78 (0.71-0.86) | 1.52E-07 | 0.84 (0.80-0.88) | 6.35E-13 |
| rs11588931 | 1 | 199,898,416 | 0.78 (0.71-0.85) | 1.77E-08 | 0.84 (0.80-0.88) | 6.52E-14 |
| rs12029406 | 1 | 199,905,828 | 0.78 (0.72-0.85) | 2.69E-08 | 0.84 (0.80-0.88) | 5.59E-14 |
| rs12024418 | 1 | 199,916,164 | 0.78 (0.72-0.86) | 4.60E-08 | 0.84 (0.80-0.88) | 9.68E-14 |
| rs4915189 | 1 | 199,916,450 | 0.78 (0.72-0.85) | 3.49E-08 | 0.84 (0.80-0.88) | 1.07E-13 |
| rs970707 | 1 | 199,920,188 | 0.78 (0.71-0.85) | 2.35E-08 | 0.83 (0.80-0.87) | 7.09E-15 |
| rs1991245 | 1 | 199,925,306 | 0.78 (0.72-0.85) | 5.62E-08 | 0.83 (0.80-0.87) | 7.83E-15 |
| rs12047611 | 1 | 199,934,748 | 0.78 (0.71-0.85) | 5.03E-08 | 0.83 (0.79-0.87) | 3.32E-15 |
| rs12726728 | 1 | 199,935,736 | 0.78 (0.71-0.85) | 6.93E-08 | 0.83 (0.79-0.87) | 5.85E-15 |
| rs10919786 | 1 | 199,946,176 | 0.78 (0.72-0.86) | 1.08E-07 | 0.83 (0.79-0.87) | 7.08E-15 |
| rs2816938 | 1 | 199,985,368 | 1.46 (1.27-1.67) | 6.39E-08 | 1.21 (1.15-1.27) | 2.13E-12 |
| rs2816941 | 1 | 199,990,779 | 1.45 (1.27-1.66) | 5.50E-08 | 1.21 (1.15-1.27) | 1.68E-12 |
| rs2816945 | 1 | 199,992,365 | 1.45 (1.27-1.66) | 8.54E-08 | 1.21 (1.15-1.27) | 1.71E-12 |
| rs2816946 | 1 | 199,993,451 | 1.45 (1.27-1.67) | 7.21E-08 | 1.21 (1.15-1.27) | 1.50E-12 |
| rs7546336 | 1 | 199,994,841 | 1.43 (1.25-1.64) | 1.50E-07 | 1.20 (1.14-1.26) | 6.57E-12 |
| rs2816949 | 1 | 199,997,778 | 1.43 (1.23-1.66) | 2.34E-06 | 1.21 (1.15-1.28) | 2.40E-12 |
| rs2816950 | 1 | 199,998,491 | 1.44 (1.24-1.68) | 1.80E-06 | 1.22 (1.15-1.28) | 2.21E-12 |
| rs2737657 | 1 | 200,001,816 | 1.43 (1.23-1.67) | 3.46E-06 | 1.22 (1.15-1.28) | 1.94E-12 |
| rs2737660 | 1 | 200,002,067 | 1.43 (1.23-1.66) | 4.46E-06 | 1.22 (1.15-1.28) | 2.37E-12 |
| rs2737661 | 1 | 200,002,068 | 1.43 (1.23-1.66) | 4.49E-06 | 1.22 (1.15-1.28) | 2.26E-12 |
| rs2821358 | 1 | 200,002,337 | 1.44 (1.24-1.68) | 2.17E-06 | 1.22 (1.15-1.29) | 1.13E-12 |
| rs3790843 | 1 | 200,010,824 | 0.76 (0.68-0.86) | 9.26E-06 | 0.83 (0.79-0.87) | 1.95E-13 |
| rs4671183 | 2 | 67,643,677 | 1.23 (1.12-1.35) | 8.25E-06 | 1.11 (1.06-1.16) | 1.26E-05 |
| rs6753785 | 2 | 111,923,630 | 1.20 (1.11-1.30) | 5.39E-06 | 1.07 (1.02-1.12) | 4.01E-03 |
| rs36018702 | 2 | 111,928,373 | 1.20 (1.11-1.30) | 4.85E-06 | 1.07 (1.02-1.12) | 3.37E-03 |
| rs4849442 | 2 | 111,934,219 | 1.20 (1.11-1.30) | 5.50E-06 | 1.07 (1.02-1.12) | 3.30E-03 |
| rs12637453 | 3 | 5,073,483 | 0.48 (0.34-0.66) | 8.14E-06 | 0.87 (0.81-0.94) | 5.46E-04 |
| rs35171303 | 3 | 5,076,614 | 0.48 (0.35-0.66) | 5.68E-06 | 0.88 (0.81-0.94) | 5.81E-04 |
| rs9854771 | 3 | 189,508,471 | 0.81 (0.73-0.89) | 8.68E-06 | 0.88 (0.84-0.92) | 2.82E-08 |
| rs1515496 | 3 | 189,508,566 | 0.81 (0.73-0.89) | 7.55E-06 | 0.88 (0.84-0.92) | 2.02E-08 |
| rs2853672 | 5 | 1,292,983 | 0.80 (0.74-0.86) | 5.63E-09 | 0.83 (0.79-0.87) | 1.89E-15 |
| rs2735940 | 5 | 1,296,486 | 0.80 (0.74-0.86) | 3.38E-09 | 0.82 (0.79-0.86) | 3.16E-16 |
| rs6866294 | 5 | 1,311,693 | 1.23 (1.13-1.33) | 8.72E-07 | 1.18 (1.12-1.23) | 3.62E-12 |
| rs35953391 | 5 | 1,312,329 | 1.44 (1.23-1.68) | 4.00E-06 | 1.21 (1.15-1.28) | 1.28E-11 |
| rs13356727 | 5 | 1,312,457 | 1.23 (1.13-1.33) | 6.53E-07 | 1.18 (1.12-1.23) | 3.25E-12 |
| rs13355267 | 5 | 1,312,935 | 1.23 (1.13-1.33) | 5.16E-07 | 1.17 (1.12-1.23) | 3.71E-12 |
| rs36115365 | 5 | 1,313,242 | 1.43 (1.23-1.67) | 5.12E-06 | 1.21 (1.14-1.28) | 1.63E-11 |
| rs28379291 | 5 | 1,313,701 | 1.21 (1.11-1.31) | 9.74E-06 | 1.18 (1.12-1.23) | 4.02E-12 |
| rs10078017 | 5 | 1,314,009 | 1.21 (1.11-1.31) | 8.90E-06 | 1.18 (1.13-1.23) | 1.96E-12 |
| rs4975615 | 5 | 1,315,343 | 1.21 (1.11-1.31) | 9.91E-06 | 1.18 (1.12-1.23) | 2.86E-12 |
| rs4975616 | 5 | 1,315,660 | 1.23 (1.14-1.33) | 3.35E-07 | 1.17 (1.12-1.23) | 2.39E-12 |
| rs13170453 | 5 | 1,317,481 | 1.57 (1.37-1.81) | 3.50E-10 | 1.22 (1.16-1.29) | 1.16E-13 |
| rs3816659 | 5 | 1,317,820 | 1.27 (1.17-1.37) | 7.55E-09 | 1.19 (1.14-1.24) | 8.29E-14 |
| rs451360 | 5 | 1,319,680 | 1.54 (1.34-1.78) | 2.83E-09 | 1.23 (1.16-1.29) | 5.78E-14 |
| rs421629 | 5 | 1,320,136 | 1.27 (1.18-1.38) | 7.52E-10 | 1.19 (1.14-1.25) | 1.25E-14 |
| rs380286 | 5 | 1,320,247 | 1.27 (1.18-1.38) | 8.46E-10 | 1.19 (1.14-1.25) | 1.17E-14 |
| rs402710 | 5 | 1,320,722 | 1.25 (1.14-1.38) | 1.74E-06 | 1.15 (1.10-1.21) | 2.91E-09 |
| rs401681 | 5 | 1,322,087 | 1.27 (1.18-1.37) | 8.38E-10 | 1.19 (1.14-1.25) | 2.10E-14 |
| rs381949 | 5 | 1,322,468 | 1.27 (1.17-1.37) | 8.59E-09 | 1.19 (1.14-1.24) | 5.54E-14 |
| rs13178866 | 5 | 1,323,212 | 1.27 (1.18-1.37) | 1.03E-09 | 1.19 (1.14-1.25) | 1.56E-14 |
| rs414965 | 5 | 1,324,121 | 1.27 (1.17-1.37) | 9.52E-09 | 1.19 (1.14-1.24) | 3.99E-14 |
| rs421284 | 5 | 1,325,590 | 1.27 (1.18-1.37) | 7.39E-10 | 1.19 (1.14-1.25) | 9.22E-15 |
| rs466502 | 5 | 1,325,767 | 1.27 (1.18-1.37) | 1.05E-09 | 1.19 (1.14-1.24) | 2.92E-14 |
| rs465498 | 5 | 1,325,803 | 1.27 (1.18-1.37) | 9.10E-10 | 1.19 (1.14-1.25) | 2.44E-14 |
| rs383009 | 5 | 1,327,851 | 1.27 (1.17-1.37) | 8.02E-09 | 1.19 (1.14-1.24) | 6.66E-14 |
| rs380145 | 5 | 1,328,897 | 1.54 (1.33-1.77) | 3.59E-09 | 1.22 (1.16-1.29) | 3.96E-13 |
| rs452932 | 5 | 1,330,253 | 1.27 (1.18-1.37) | 1.04E-09 | 1.19 (1.14-1.25) | 2.14E-14 |
| rs452384 | 5 | 1,330,840 | 1.27 (1.18-1.37) | 1.06E-09 | 1.19 (1.14-1.25) | 2.32E-14 |
| rs370348 | 5 | 1,331,219 | 1.27 (1.18-1.37) | 9.23E-10 | 1.19 (1.14-1.25) | 2.07E-14 |
| rs2447853 | 5 | 1,333,077 | 1.28 (1.18-1.38) | 4.19E-10 | 1.19 (1.14-1.25) | 1.12E-14 |
| rs457130 | 5 | 1,336,178 | 1.26 (1.16-1.37) | 1.34E-08 | 1.19 (1.14-1.24) | 5.81E-14 |
| rs467095 | 5 | 1,336,221 | 1.27 (1.18-1.37) | 1.14E-09 | 1.19 (1.14-1.25) | 2.08E-14 |
| rs455433 | 5 | 1,336,243 | 1.27 (1.18-1.37) | 1.24E-09 | 1.19 (1.14-1.25) | 2.47E-14 |
| rs460073 | 5 | 1,336,459 | 1.27 (1.18-1.37) | 1.27E-09 | 1.19 (1.14-1.25) | 2.22E-14 |
| rs462608 | 5 | 1,336,626 | 1.26 (1.17-1.37) | 9.67E-09 | 1.19 (1.14-1.24) | 5.81E-14 |
| rs456366 | 5 | 1,337,070 | 1.27 (1.18-1.37) | 1.04E-09 | 1.19 (1.14-1.25) | 1.98E-14 |
| rs459961 | 5 | 1,337,106 | 1.27 (1.18-1.37) | 1.10E-09 | 1.19 (1.14-1.25) | 2.36E-14 |
| rs31484 | 5 | 1,337,906 | 1.27 (1.18-1.37) | 1.01E-09 | 1.19 (1.14-1.25) | 2.04E-14 |
| rs31487 | 5 | 1,341,101 | 1.27 (1.17-1.37) | 2.00E-09 | 1.19 (1.14-1.24) | 2.88E-14 |
| rs31489 | 5 | 1,342,714 | 1.28 (1.18-1.38) | 3.98E-09 | 1.20 (1.14-1.25) | 4.62E-15 |
| rs31490 | 5 | 1,344,458 | 1.28 (1.18-1.38) | 5.21E-10 | 1.20 (1.15-1.25) | 2.38E-15 |
| rs27996 | 5 | 1,345,474 | 1.23 (1.14-1.34) | 1.36E-07 | 1.17 (1.12-1.22) | 1.29E-11 |
| rs27071 | 5 | 1,346,081 | 1.39 (1.22-1.57) | 3.85E-07 | 1.18 (1.12-1.24) | 4.02E-10 |
| rs27070 | 5 | 1,346,303 | 1.24 (1.15-1.34) | 7.35E-08 | 1.18 (1.12-1.23) | 3.00E-12 |
| rs27069 | 5 | 1,347,128 | 1.23 (1.13-1.33) | 8.18E-07 | 1.16 (1.11-1.21) | 4.26E-10 |
| rs27068 | 5 | 1,347,239 | 1.39 (1.23-1.58) | 2.56E-07 | 1.18 (1.12-1.24) | 9.39E-10 |
| rs37011 | 5 | 1,348,798 | 1.25 (1.15-1.35) | 4.20E-08 | 1.18 (1.12-1.23) | 3.96E-12 |
| rs37010 | 5 | 1,349,535 | 1.26 (1.16-1.36) | 1.87E-08 | 1.18 (1.12-1.23) | 4.83E-12 |
| rs37009 | 5 | 1,350,339 | 1.24 (1.15-1.35) | 7.56E-08 | 1.17 (1.11-1.22) | 5.73E-11 |
| rs40182 | 5 | 1,350,397 | 1.24 (1.15-1.35) | 8.40E-08 | 1.16 (1.11-1.22) | 6.71E-11 |
| rs37007 | 5 | 1,352,372 | 1.26 (1.16-1.36) | 2.41E-08 | 1.17 (1.12-1.23) | 9.58E-12 |
| rs37004 | 5 | 1,356,684 | 1.51 (1.30-1.74) | 3.72E-08 | 1.21 (1.14-1.28) | 1.28E-11 |
| rs2937630 | 5 | 1,376,326 | 1.41 (1.21-1.65) | 9.86E-06 | 1.13 (1.07-1.19) | 2.50E-05 |
| rs2927670 | 5 | 1,377,075 | 1.41 (1.22-1.65) | 7.98E-06 | 1.13 (1.07-1.19) | 2.08E-05 |
| rs2963259 | 5 | 1,377,172 | 1.41 (1.21-1.65) | 8.22E-06 | 1.12 (1.06-1.19) | 2.67E-05 |
| rs78233656 | 6 | 61,951,962 | 6.10 (3.05-12.24) | 3.43E-07 | 1.00 (0.94-1.07) | 9.55E-01 |
| rs10272516 | 7 | 40,837,737 | 0.84 (0.78-0.91) | 9.27E-06 | 0.89 (0.85-0.93) | 8.32E-07 |
| rs1203573 | 7 | 40,841,572 | 0.84 (0.78-0.91) | 6.56E-06 | 0.89 (0.85-0.94) | 1.02E-06 |
| rs13230202 | 7 | 40,865,219 | 0.73 (0.64-0.83) | 1.53E-06 | 0.86 (0.82-0.91) | 1.72E-08 |
| rs17688583 | 7 | 40,865,696 | 0.73 (0.65-0.83) | 1.72E-06 | 0.86 (0.82-0.91) | 1.25E-08 |
| rs1012568 | 7 | 40,866,235 | 0.73 (0.65-0.83) | 2.07E-06 | 0.86 (0.82-0.91) | 1.72E-08 |
| rs1012567 | 7 | 40,866,242 | 0.73 (0.64-0.83) | 1.74E-06 | 0.86 (0.82-0.91) | 1.73E-08 |
| rs17688601 | 7 | 40,866,663 | 0.73 (0.64-0.83) | 1.69E-06 | 0.86 (0.82-0.91) | 1.19E-08 |
| rs10279715 | 7 | 40,870,935 | 0.82 (0.76-0.89) | 1.30E-06 | 0.88 (0.84-0.92) | 1.12E-08 |
| rs1733483 | 7 | 40,873,221 | 0.84 (0.78-0.91) | 4.59E-06 | 0.89 (0.85-0.93) | 4.62E-07 |
| rs17621345 | 7 | 40,875,192 | 0.74 (0.65-0.84) | 7.00E-06 | 0.87 (0.82-0.91) | 6.64E-08 |
| rs12701838 | 7 | 40,877,473 | 0.72 (0.63-0.81) | 2.22E-07 | 0.86 (0.82-0.90) | 3.55E-09 |
| rs4731300 | 7 | 125,773,957 | 0.82 (0.75-0.89) | 7.69E-06 | 0.91 (0.87-0.96) | 9.77E-05 |
| rs4626538 | 7 | 129,413,188 | 0.84 (0.78-0.91) | 8.60E-06 | 0.93 (0.89-0.97) | 1.61E-03 |
| rs11978468 | 7 | 130,671,425 | 0.79 (0.71-0.87) | 5.75E-06 | 0.90 (0.86-0.94) | 1.22E-05 |
| rs6467327 | 7 | 130,671,815 | 0.78 (0.70-0.86) | 1.64E-06 | 0.90 (0.85-0.94) | 7.78E-06 |
| rs4731722 | 7 | 130,672,847 | 0.76 (0.67-0.85) | 1.54E-06 | 0.89 (0.84-0.93) | 1.45E-06 |
| rs6467328 | 7 | 130,673,153 | 0.75 (0.67-0.84) | 6.24E-07 | 0.88 (0.84-0.93) | 4.73E-07 |
| rs10261974 | 7 | 130,673,625 | 0.74 (0.66-0.83) | 4.06E-07 | 0.88 (0.84-0.92) | 4.24E-07 |
| rs17165346 | 7 | 130,674,154 | 0.74 (0.66-0.84) | 1.24E-06 | 0.87 (0.83-0.92) | 1.21E-07 |
| rs57938547 | 7 | 130,674,215 | 0.75 (0.67-0.85) | 4.43E-06 | 0.88 (0.83-0.92) | 3.51E-07 |
| rs4731724 | 7 | 130,674,523 | 0.74 (0.66-0.83) | 6.08E-07 | 0.88 (0.84-0.93) | 6.22E-07 |
| rs10251370 | 7 | 130,675,225 | 0.72 (0.64-0.82) | 3.69E-07 | 0.88 (0.83-0.92) | 6.61E-07 |
| rs7008921 | 8 | 5,424,408 | 2.30 (1.59-3.32) | 8.85E-06 | 1.06 (0.98-1.14) | 1.60E-01 |
| rs663859 | 9 | 106,779,440 | 1.24 (1.14-1.36) | 2.35E-06 | 1.13 (1.08-1.19) | 9.82E-08 |
| rs10991043 | 9 | 106,797,388 | 1.24 (1.14-1.36) | 2.69E-06 | 1.14 (1.08-1.19) | 6.13E-08 |
| rs1888028 | 9 | 106,810,372 | 1.24 (1.14-1.36) | 2.48E-06 | 1.14 (1.08-1.19) | 7.65E-08 |
| rs2073826 | 9 | 136,136,963 | 0.81 (0.75-0.89) | 3.17E-06 | 0.86 (0.82-0.90) | 1.10E-10 |
| rs687621 | 9 | 136,137,065 | 1.25 (1.14-1.37) | 2.27E-06 | 1.25 (1.20-1.31) | 4.26E-21 |
| rs687289 | 9 | 136,137,106 | 1.25 (1.14-1.37) | 2.01E-06 | 1.26 (1.20-1.32) | 2.80E-21 |
| rs2073827 | 9 | 136,137,133 | 0.81 (0.75-0.89) | 3.32E-06 | 0.86 (0.82-0.90) | 1.10E-10 |
| rs2073828 | 9 | 136,137,140 | 0.81 (0.75-0.89) | 3.36E-06 | 0.86 (0.82-0.90) | 1.16E-10 |
| rs8176690 | 9 | 136,138,317 | 0.81 (0.75-0.89) | 3.95E-06 | 0.86 (0.82-0.90) | 1.73E-10 |
| rs657152 | 9 | 136,139,265 | 1.23 (1.13-1.35) | 2.61E-06 | 1.23 (1.17-1.29) | 3.29E-18 |
| rs8176681 | 9 | 136,139,754 | 0.82 (0.75-0.89) | 5.41E-06 | 0.86 (0.82-0.90) | 2.51E-10 |
| rs514659 | 9 | 136,142,203 | 1.24 (1.13-1.36) | 3.58E-06 | 1.25 (1.19-1.31) | 2.63E-20 |
| rs644234 | 9 | 136,142,217 | 1.23 (1.13-1.34) | 3.86E-06 | 1.23 (1.17-1.28) | 8.71E-18 |
| rs643434 | 9 | 136,142,355 | 1.23 (1.13-1.34) | 3.80E-06 | 1.23 (1.17-1.28) | 7.91E-18 |
| rs613534 | 9 | 136,143,120 | 1.23 (1.13-1.34) | 3.57E-06 | 1.23 (1.17-1.29) | 7.53E-18 |
| rs543968 | 9 | 136,143,121 | 1.23 (1.13-1.34) | 3.57E-06 | 1.23 (1.17-1.29) | 7.53E-18 |
| rs544873 | 9 | 136,143,212 | 1.23 (1.13-1.34) | 3.57E-06 | 1.23 (1.17-1.29) | 7.53E-18 |
| rs545971 | 9 | 136,143,372 | 1.24 (1.13-1.36) | 3.71E-06 | 1.25 (1.19-1.31) | 2.46E-20 |
| rs612169 | 9 | 136,143,442 | 1.24 (1.13-1.36) | 3.44E-06 | 1.25 (1.19-1.31) | 2.32E-20 |
| rs597988 | 9 | 136,144,284 | 1.24 (1.14-1.36) | 3.35E-06 | 1.25 (1.19-1.31) | 2.12E-20 |
| rs8176663 | 9 | 136,144,427 | 1.24 (1.13-1.36) | 3.71E-06 | 1.25 (1.19-1.31) | 2.46E-20 |
| rs491626 | 9 | 136,144,873 | 1.24 (1.13-1.36) | 3.71E-06 | 1.25 (1.19-1.31) | 2.46E-20 |
| rs492488 | 9 | 136,144,960 | 1.24 (1.13-1.36) | 3.71E-06 | 1.25 (1.19-1.31) | 2.46E-20 |
| rs493246 | 9 | 136,144,994 | 1.24 (1.13-1.36) | 3.71E-06 | 1.25 (1.19-1.31) | 2.46E-20 |
| rs494242 | 9 | 136,145,118 | 1.23 (1.13-1.34) | 3.95E-06 | 1.23 (1.17-1.28) | 8.69E-18 |
| rs495203 | 9 | 136,145,240 | 1.24 (1.13-1.36) | 3.71E-06 | 1.25 (1.19-1.31) | 2.46E-20 |
| rs582118 | 9 | 136,145,471 | 1.25 (1.14-1.37) | 3.23E-06 | 1.25 (1.19-1.31) | 2.13E-20 |
| rs582094 | 9 | 136,145,484 | 1.24 (1.13-1.36) | 5.06E-06 | 1.25 (1.19-1.31) | 4.31E-20 |
| rs2769071 | 9 | 136,145,974 | 1.24 (1.13-1.36) | 3.60E-06 | 1.25 (1.19-1.31) | 3.01E-20 |
| rs677355 | 9 | 136,146,046 | 1.25 (1.14-1.37) | 2.68E-06 | 1.25 (1.19-1.31) | 1.67E-20 |
| rs676996 | 9 | 136,146,077 | 1.25 (1.14-1.37) | 3.05E-06 | 1.25 (1.19-1.31) | 2.05E-20 |
| rs676457 | 9 | 136,146,227 | 1.25 (1.14-1.37) | 3.05E-06 | 1.25 (1.19-1.31) | 2.05E-20 |
| rs527210 | 9 | 136,146,431 | 1.25 (1.14-1.37) | 2.30E-06 | 1.25 (1.19-1.31) | 1.34E-20 |
| rs674302 | 9 | 136,146,664 | 1.25 (1.14-1.37) | 3.05E-06 | 1.25 (1.19-1.31) | 2.33E-20 |
| rs554833 | 9 | 136,147,160 | 1.25 (1.14-1.37) | 3.09E-06 | 1.25 (1.19-1.31) | 2.04E-20 |
| rs660340 | 9 | 136,147,553 | 0.79 (0.73-0.86) | 1.44E-08 | 0.89 (0.85-0.93) | 2.81E-07 |
| rs581107 | 9 | 136,147,702 | 0.79 (0.73-0.86) | 1.42E-08 | 0.89 (0.85-0.93) | 2.76E-07 |
| rs659104 | 9 | 136,147,823 | 0.79 (0.73-0.86) | 1.26E-08 | 0.89 (0.85-0.93) | 2.77E-07 |
| rs647800 | 9 | 136,148,000 | 0.79 (0.73-0.86) | 1.09E-07 | 0.90 (0.86-0.94) | 3.85E-06 |
| rs473533 | 9 | 136,148,035 | 0.79 (0.73-0.86) | 1.28E-08 | 0.89 (0.85-0.93) | 2.65E-07 |
| rs475419 | 9 | 136,148,231 | 0.79 (0.72-0.85) | 1.14E-08 | 0.89 (0.85-0.93) | 2.49E-07 |
| rs476410 | 9 | 136,148,368 | 0.79 (0.73-0.86) | 1.30E-08 | 0.89 (0.85-0.93) | 2.59E-07 |
| rs645982 | 9 | 136,148,409 | 0.79 (0.72-0.85) | 1.20E-08 | 0.89 (0.85-0.93) | 2.42E-07 |
| rs500498 | 9 | 136,148,647 | 0.79 (0.72-0.85) | 1.09E-08 | 0.89 (0.85-0.93) | 2.61E-07 |
| rs500499 | 9 | 136,148,648 | 0.79 (0.73-0.85) | 1.19E-08 | 0.89 (0.85-0.93) | 2.78E-07 |
| rs505922 | 9 | 136,149,229 | 1.25 (1.14-1.37) | 2.95E-06 | 1.25 (1.19-1.31) | 1.14E-20 |
| rs529565 | 9 | 136,149,500 | 1.25 (1.14-1.37) | 3.26E-06 | 1.25 (1.19-1.31) | 2.25E-20 |
| rs630510 | 9 | 136,149,581 | 0.76 (0.70-0.82) | 9.64E-12 | 0.85 (0.81-0.89) | 3.74E-13 |
| rs630014 | 9 | 136,149,722 | 0.76 (0.70-0.82) | 9.64E-12 | 0.85 (0.81-0.89) | 3.74E-13 |
| rs616154 | 9 | 136,150,466 | 0.77 (0.71-0.83) | 3.14E-11 | 0.85 (0.81-0.89) | 6.35E-13 |
| rs559723 | 9 | 136,150,484 | 0.76 (0.71-0.83) | 1.74E-11 | 0.85 (0.81-0.89) | 3.89E-13 |
| rs633862 | 9 | 136,155,444 | 0.80 (0.74-0.87) | 1.23E-07 | 0.89 (0.85-0.93) | 7.19E-07 |
| rs7985481 | 13 | 28,466,090 | 1.26 (1.16-1.37) | 9.07E-08 | 1.15 (1.10-1.20) | 3.21E-09 |
| rs9581931 | 13 | 28,468,222 | 1.26 (1.16-1.37) | 7.83E-08 | 1.15 (1.10-1.20) | 3.52E-09 |
| rs4424773 | 13 | 28,470,544 | 1.26 (1.16-1.37) | 3.60E-08 | 1.15 (1.10-1.20) | 1.44E-09 |
| rs9554193 | 13 | 28,471,366 | 1.26 (1.16-1.37) | 3.23E-08 | 1.15 (1.10-1.21) | 8.62E-10 |
| rs7995917 | 13 | 28,472,724 | 1.26 (1.16-1.37) | 3.74E-08 | 1.15 (1.10-1.21) | 9.78E-10 |
| rs7993114 | 13 | 28,476,277 | 1.25 (1.16-1.36) | 5.86E-08 | 1.15 (1.10-1.21) | 7.31E-10 |
| rs11618581 | 13 | 28,476,911 | 1.25 (1.15-1.36) | 6.67E-08 | 1.15 (1.10-1.20) | 8.30E-10 |
| rs9554197 | 13 | 28,476,978 | 1.25 (1.15-1.36) | 6.67E-08 | 1.15 (1.10-1.20) | 8.30E-10 |
| rs11618036 | 13 | 28,477,058 | 1.25 (1.15-1.36) | 6.67E-08 | 1.15 (1.10-1.20) | 8.30E-10 |
| rs11618832 | 13 | 28,477,111 | 1.25 (1.15-1.36) | 6.67E-08 | 1.15 (1.10-1.20) | 8.30E-10 |
| rs11616678 | 13 | 28,477,117 | 1.25 (1.15-1.36) | 6.67E-08 | 1.15 (1.10-1.20) | 8.30E-10 |
| rs11618052 | 13 | 28,477,149 | 1.25 (1.15-1.36) | 6.67E-08 | 1.15 (1.10-1.20) | 8.30E-10 |
| rs9579127 | 13 | 28,477,268 | 1.25 (1.15-1.36) | 6.67E-08 | 1.15 (1.10-1.20) | 8.30E-10 |
| rs7999100 | 13 | 28,477,401 | 1.25 (1.15-1.36) | 6.67E-08 | 1.15 (1.10-1.20) | 8.30E-10 |
| rs8000004 | 13 | 28,477,590 | 1.25 (1.15-1.36) | 7.06E-08 | 1.15 (1.10-1.20) | 8.74E-10 |
| rs9579128 | 13 | 28,481,938 | 1.25 (1.16-1.36) | 6.80E-08 | 1.15 (1.10-1.20) | 9.09E-10 |
| rs2297316 | 13 | 28,490,211 | 1.27 (1.17-1.38) | 1.39E-08 | 1.16 (1.11-1.21) | 2.24E-10 |
| rs9581943 | 13 | 28,493,997 | 1.28 (1.17-1.39) | 1.02E-08 | 1.15 (1.10-1.21) | 5.07E-10 |
| rs8002057 | 13 | 73,895,877 | 1.35 (1.22-1.49) | 2.45E-09 | 1.21 (1.15-1.27) | 2.91E-15 |
| rs9564966 | 13 | 73,896,221 | 1.35 (1.22-1.49) | 1.74E-09 | 1.21 (1.16-1.27) | 1.74E-15 |
| rs9543321 | 13 | 73,896,659 | 1.35 (1.23-1.49) | 1.50E-09 | 1.21 (1.16-1.27) | 1.62E-15 |
| rs9564967 | 13 | 73,901,152 | 1.35 (1.23-1.49) | 1.30E-09 | 1.21 (1.16-1.27) | 1.54E-15 |
| rs7330393 | 13 | 73,901,905 | 1.36 (1.23-1.49) | 1.13E-09 | 1.21 (1.16-1.27) | 1.36E-15 |
| rs9573163 | 13 | 73,908,846 | 1.35 (1.23-1.49) | 1.51E-09 | 1.21 (1.16-1.27) | 1.13E-15 |
| rs9573164 | 13 | 73,909,020 | 1.35 (1.23-1.49) | 1.44E-09 | 1.22 (1.16-1.27) | 6.85E-16 |
| rs9564968 | 13 | 73,909,322 | 1.35 (1.23-1.49) | 1.20E-09 | 1.21 (1.16-1.27) | 1.32E-15 |
| rs4885093 | 13 | 73,910,026 | 1.39 (1.28-1.52) | 2.15E-13 | 1.25 (1.19-1.31) | 1.20E-20 |
| rs9543324 | 13 | 73,914,797 | 1.39 (1.24-1.56) | 3.89E-08 | 1.21 (1.15-1.28) | 2.70E-14 |
| rs2038790 | 13 | 73,915,644 | 1.40 (1.28-1.53) | 1.01E-13 | 1.24 (1.19-1.30) | 1.57E-20 |
| rs9543325 | 13 | 73,916,628 | 1.40 (1.28-1.53) | 1.07E-13 | 1.24 (1.19-1.30) | 1.49E-20 |
| rs9573165 | 13 | 73,917,375 | 1.40 (1.28-1.53) | 9.22E-14 | 1.25 (1.19-1.30) | 1.26E-20 |
| rs2147512 | 13 | 73,924,784 | 1.39 (1.27-1.52) | 5.83E-13 | 1.24 (1.18-1.30) | 1.26E-19 |
| rs9573166 | 13 | 73,926,086 | 1.41 (1.29-1.53) | 5.64E-15 | 1.26 (1.20-1.32) | 5.78E-22 |
| rs12427746 | 13 | 73,930,590 | 1.33 (1.18-1.50) | 3.69E-06 | 1.17 (1.11-1.23) | 6.77E-10 |
| rs4888360 | 16 | 75,254,584 | 1.38 (1.24-1.53) | 5.31E-09 | 1.14 (1.09-1.20) | 7.88E-08 |
| rs10871306 | 16 | 75,254,680 | 1.37 (1.24-1.52) | 2.41E-09 | 1.14 (1.08-1.19) | 2.01E-07 |
| rs8048784 | 16 | 75,254,862 | 1.38 (1.24-1.53) | 4.74E-09 | 1.14 (1.09-1.20) | 7.46E-08 |
| rs8055167 | 16 | 75,254,889 | 1.38 (1.24-1.54) | 3.99E-09 | 1.14 (1.09-1.20) | 6.28E-08 |
| rs8051037 | 16 | 75,255,062 | 1.38 (1.24-1.54) | 3.85E-09 | 1.14 (1.09-1.20) | 6.95E-08 |
| rs8051363 | 16 | 75,255,217 | 1.46 (1.30-1.65) | 1.70E-10 | 1.18 (1.12-1.24) | 6.31E-11 |
| rs9652674 | 16 | 75,255,473 | 1.38 (1.24-1.53) | 4.92E-09 | 1.14 (1.09-1.20) | 6.88E-08 |
| rs9652665 | 16 | 75,255,516 | 1.37 (1.24-1.53) | 5.70E-09 | 1.14 (1.09-1.20) | 8.17E-08 |
| rs8045196 | 16 | 75,256,360 | 1.46 (1.29-1.65) | 2.66E-09 | 1.17 (1.11-1.23) | 1.81E-09 |
| rs11645191 | 16 | 75,274,980 | 1.25 (1.14-1.39) | 9.04E-06 | 1.12 (1.07-1.17) | 4.77E-06 |
| rs1035539 | 16 | 75,276,775 | 1.26 (1.14-1.39) | 4.36E-06 | 1.12 (1.07-1.17) | 3.90E-06 |
| rs4888369 | 16 | 75,307,277 | 1.59 (1.30-1.95) | 6.98E-06 | 1.19 (1.12-1.27) | 1.50E-08 |
| rs4888370 | 16 | 75,308,230 | 1.59 (1.30-1.95) | 6.57E-06 | 1.19 (1.12-1.27) | 1.43E-08 |
| rs6564245 | 16 | 75,308,899 | 1.59 (1.30-1.94) | 7.61E-06 | 1.19 (1.12-1.26) | 2.17E-08 |
| rs7187263 | 16 | 75,308,978 | 1.59 (1.30-1.95) | 6.43E-06 | 1.19 (1.12-1.26) | 2.22E-08 |
| rs4887812 | 16 | 75,310,052 | 1.59 (1.30-1.94) | 7.62E-06 | 1.19 (1.12-1.27) | 1.97E-08 |
| rs4887814 | 16 | 75,318,041 | 1.59 (1.29-1.95) | 9.67E-06 | 1.19 (1.12-1.27) | 2.50E-08 |
| rs12449177 | 16 | 75,332,603 | 1.38 (1.20-1.59) | 6.88E-06 | 1.14 (1.08-1.20) | 8.40E-07 |
| rs11149813 | 16 | 75,332,655 | 1.38 (1.20-1.59) | 6.50E-06 | 1.14 (1.08-1.21) | 7.20E-07 |
| rs3784937 | 16 | 75,337,591 | 1.40 (1.22-1.62) | 2.33E-06 | 1.15 (1.09-1.21) | 4.23E-07 |
| rs10781977 | 16 | 75,342,932 | 1.40 (1.22-1.61) | 2.48E-06 | 1.14 (1.09-1.21) | 5.56E-07 |
| rs889446 | 16 | 75,344,787 | 1.40 (1.22-1.61) | 2.38E-06 | 1.15 (1.09-1.21) | 4.26E-07 |
| rs12445813 | 16 | 75,348,622 | 1.41 (1.22-1.62) | 2.03E-06 | 1.15 (1.09-1.21) | 2.26E-07 |
| rs12445188 | 16 | 75,348,657 | 1.41 (1.22-1.62) | 2.00E-06 | 1.15 (1.09-1.21) | 2.60E-07 |
| rs7199680 | 16 | 75,355,957 | 1.41 (1.22-1.62) | 1.86E-06 | 1.15 (1.09-1.21) | 2.24E-07 |
| rs1017239 | 16 | 75,358,207 | 1.40 (1.22-1.62) | 2.51E-06 | 1.15 (1.09-1.21) | 2.32E-07 |
| rs2032926 | 16 | 75,358,910 | 1.41 (1.22-1.63) | 1.91E-06 | 1.15 (1.09-1.21) | 2.99E-07 |
| rs8051785 | 16 | 75,361,036 | 1.39 (1.21-1.60) | 4.78E-06 | 1.15 (1.09-1.21) | 5.37E-07 |
| rs11149816 | 16 | 75,361,767 | 1.39 (1.21-1.60) | 4.14E-06 | 1.15 (1.09-1.21) | 4.77E-07 |
| rs7192155 | 16 | 75,382,194 | 1.37 (1.20-1.57) | 3.85E-06 | 1.15 (1.09-1.21) | 1.65E-07 |
| rs7202083 | 16 | 75,383,727 | 1.70 (1.35-2.13) | 5.56E-06 | 1.23 (1.16-1.31) | 2.16E-10 |
| rs3851736 | 16 | 75,387,332 | 1.59 (1.29-1.95) | 9.37E-06 | 1.19 (1.12-1.26) | 2.89E-08 |
| rs8051611 | 16 | 75,388,933 | 1.59 (1.29-1.95) | 9.50E-06 | 1.19 (1.12-1.26) | 3.72E-08 |
| rs1074961 | 16 | 75,389,484 | 1.59 (1.30-1.95) | 7.84E-06 | 1.19 (1.12-1.26) | 4.08E-08 |
| rs9934007 | 16 | 75,390,532 | 1.43 (1.24-1.64) | 5.48E-07 | 1.16 (1.10-1.22) | 8.31E-08 |
| rs8048677 | 16 | 75,392,028 | 1.59 (1.30-1.95) | 7.89E-06 | 1.19 (1.12-1.26) | 3.57E-08 |
| rs2059257 | 16 | 75,396,376 | 1.60 (1.30-1.96) | 7.39E-06 | 1.18 (1.11-1.26) | 6.42E-08 |
| rs6564256 | 16 | 75,397,509 | 1.59 (1.30-1.95) | 7.86E-06 | 1.19 (1.12-1.26) | 4.00E-08 |
| rs8055609 | 16 | 75,402,088 | 1.60 (1.31-1.97) | 5.75E-06 | 1.19 (1.12-1.26) | 2.59E-08 |
| rs11645691 | 16 | 75,409,235 | 1.42 (1.24-1.64) | 6.65E-07 | 1.16 (1.10-1.22) | 6.65E-08 |
| rs8049611 | 16 | 75,409,474 | 1.60 (1.30-1.96) | 6.55E-06 | 1.18 (1.11-1.26) | 5.65E-08 |
| rs8054586 | 16 | 75,410,705 | 1.59 (1.30-1.95) | 9.09E-06 | 1.19 (1.12-1.26) | 2.97E-08 |
| rs8046949 | 16 | 75,420,609 | 1.44 (1.25-1.67) | 5.47E-07 | 1.15 (1.09-1.21) | 1.98E-07 |
| rs4888396 | 16 | 75,420,750 | 1.37 (1.19-1.57) | 7.67E-06 | 1.14 (1.08-1.20) | 6.46E-07 |
| rs4888397 | 16 | 75,421,082 | 1.43 (1.24-1.65) | 1.01E-06 | 1.15 (1.09-1.21) | 3.78E-07 |
| rs4888398 | 16 | 75,421,164 | 1.43 (1.24-1.65) | 1.01E-06 | 1.15 (1.09-1.21) | 4.06E-07 |
| rs8048816 | 16 | 75,422,201 | 1.44 (1.25-1.66) | 7.72E-07 | 1.16 (1.10-1.22) | 5.32E-08 |
| rs9989421 | 16 | 75,422,584 | 1.44 (1.25-1.67) | 6.95E-07 | 1.16 (1.10-1.23) | 4.11E-08 |
| rs766522 | 16 | 75,424,110 | 1.44 (1.25-1.66) | 7.84E-07 | 1.16 (1.10-1.22) | 5.24E-08 |
| rs247437 | 16 | 75,431,275 | 1.58 (1.29-1.94) | 9.47E-06 | 1.18 (1.11-1.25) | 1.06E-07 |
| rs12446903 | 16 | 75,438,761 | 1.43 (1.24-1.65) | 1.26E-06 | 1.15 (1.09-1.22) | 1.42E-07 |
| rs2865527 | 16 | 75,456,726 | 1.42 (1.24-1.64) | 9.37E-07 | 1.15 (1.09-1.21) | 2.43E-07 |
| rs247442 | 16 | 75,464,621 | 1.70 (1.35-2.13) | 5.89E-06 | 1.22 (1.15-1.31) | 7.54E-10 |
| rs7201989 | 16 | 75,471,499 | 1.39 (1.21-1.61) | 5.36E-06 | 1.15 (1.09-1.22) | 1.57E-07 |
| rs2242405 | 16 | 75,481,771 | 1.45 (1.25-1.67) | 7.92E-07 | 1.16 (1.10-1.22) | 1.36E-07 |
| rs4887827 | 16 | 75,482,575 | 1.44 (1.25-1.67) | 8.75E-07 | 1.16 (1.09-1.22) | 1.43E-07 |
| rs9928232 | 16 | 75,486,347 | 1.45 (1.25-1.68) | 6.12E-07 | 1.16 (1.10-1.22) | 7.01E-08 |
| rs8046416 | 16 | 75,487,758 | 1.46 (1.26-1.69) | 6.60E-07 | 1.16 (1.10-1.23) | 4.59E-08 |
| rs4887828 | 16 | 75,488,619 | 1.46 (1.26-1.69) | 5.44E-07 | 1.16 (1.10-1.23) | 4.22E-08 |
| rs4887830 | 16 | 75,489,026 | 1.46 (1.26-1.69) | 5.58E-07 | 1.16 (1.10-1.23) | 4.83E-08 |
| rs2665321 | 16 | 86,333,173 | 1.20 (1.11-1.30) | 5.35E-06 | 1.10 (1.05-1.15) | 2.38E-05 |
| rs2063341 | 16 | 86,333,623 | 1.20 (1.11-1.30) | 5.59E-06 | 1.10 (1.06-1.16) | 1.89E-05 |
| rs2696827 | 16 | 86,333,728 | 1.20 (1.11-1.30) | 4.99E-06 | 1.11 (1.06-1.16) | 1.53E-05 |
| rs7188112 | 16 | 86,334,618 | 1.21 (1.12-1.31) | 9.33E-07 | 1.11 (1.06-1.16) | 7.43E-06 |
| rs7200646 | 16 | 86,335,351 | 1.25 (1.14-1.38) | 6.43E-06 | 1.11 (1.06-1.16) | 1.20E-05 |
| rs67622136 | 17 | 43,452,519 | 0.50 (0.37-0.67) | 6.02E-06 | 0.91 (0.84-0.98) | 1.00E-02 |
| rs147904962 | 17 | 43,454,537 | 0.50 (0.37-0.67) | 4.73E-06 | 0.91 (0.85-0.98) | 9.29E-03 |
| rs9894188 | 17 | 46,044,031 | 1.23 (1.12-1.34) | 7.82E-06 | 1.09 (1.04-1.14) | 4.89E-04 |

OR: odds ratio; CI: confidence interval.

**Supplementary Table 2**. Associations of the 6 SNPs with PDAC risk under additive and recessive genetic models.

| **SNP** | **Locus** | **Position**  **(hg38)** | **Major/**  **minor** **allele** | **MAF** | **Closest gene** | **Phase** | **Recessive model** | | **Additive model** | | |  |
| --- | --- | --- | --- | --- | --- | --- | --- | --- | --- | --- | --- | --- |
|  |  |  |  |  |  |  | **OR (95% CI)** | **p** | | **OR (95% CI)** | **p** | |
| rs2066357 | 1p22.3 | 85,505,674 | G/A | 0.28 | *DDAH1* | Discovery | 0.78 (0.70-0.87) | 9.70x10^-6^ | | 0.93 (0.88-0.97) | 1.89x10^-3^ | |
|  |  |  |  | 0.31 |  | Replication | 1.26 (1.04-1.52) | 0.02 | | 1.08 (0.99-1.17) | 0.08 | |
|  |  |  |  | 0.29 |  | Meta* | 1.05 (0.76-1.45) | 0.76 | | 1.01 (0.92-1.12) | 0.82 | |
|  |  |  |  |  |  | Meta (MI)^#^ | 1.04 (0.77-1.42) | 0.79 | | 1.02 (0.91-1.15) | 0.72 | |
| rs1339571 | 1q25.3 | 184,598,799 | G/C | 0.18 | *C1orf21* | Discovery | 0.67 (0.56-0.80) | 7.25x10^-6^ | | 0.93 (0.88-0.99) | 0.02 | |
|  |  |  |  | 0.18 |  | Replication | 1.03 (0.78-1.38) | 0.82 | | 0.97 (0.89-1.07) | 0.62 | |
|  |  |  |  | 0.19 |  | Meta | 0.85 (0.79-0.92) | 1.92x10^-5^ | | 0.97 (0.95-1.00) | 0.02 | |
|  |  |  |  |  |  | Meta (MI)^#^ | 0.85 (0.79-0.92) | 2.02x10^-5^ | | 0.97 (0.95-1.00) | 0.02 | |
| rs36018702 | 2q13 | 111,170,796 | C/A | 0.45 | *BCL2L11* | Discovery | 1.20 (1.11-1.30) | 4.85x10^-6^ | | 1.07 (1.02-1.12) | 3.37x10^-3^ | |
|  |  |  |  | 0.45 |  | Replication | 0.96 (0.85-1.09) | 0.56 | | 1.01 (0.94-1.09) | 0.74 | |
|  |  |  |  | 0.49 |  | Meta | 1.07 (1.04-1.11) | 2.32x10^-5^ | | 1.03 (1.01-1.05) | 3.58x10^-3^ | |
|  |  |  |  |  |  | Meta (MI)^#^ | 1.08 (1.04-1.11) | 1.92x10^-5^ | | 1.03 (1.01-1.04) | 3.78x10^-3^ | |
| rs4626538 | 7q32.2 | 129,773,348 | T/G | 0.49 | *MIR96* | Discovery | 0.84 (0.78-0.91) | 8.60x10^-6^ | | 0.93 (0.89-0.97) | 1.61x10^-3^ | |
|  |  |  |  | 0.48 |  | Replication | 0.93 (0.82-1.06) | 0.26 | | 0.97 (0.90-1.04) | 0.38 | |
|  |  |  |  | 0.47 |  | Meta | 0.93 (0.90-0.96) | 4.42x10^-6^ | | 0.97 (0.95-0.99) | 1.06x10^-3^ | |
|  |  |  |  |  |  | Meta (MI)^#^ | 0.93 (0.90-0.96) | 6.21x10^-6^ | | 0.97 (0.95-0.99) | 1.22x10^-3^ | |
| rs7008921 | 8p23.2 | 5,566,886 | T/C | 0.09 | *RP5-991O23.1* | Discovery | 2.30 (1.59-3.32) | 8.85x10^-6^ | | 1.06 (0.98-1.14) | 0.16 | |
|  |  |  |  | 0.10 |  | Replication | 1.17 (0.63-2.15) | 0.62 | | 1.15 (0.84-1.56) | 0.02 | |
|  |  |  |  | 0.10 |  | Meta | 1.42 (1.21-1.65) | 9.68x10^-6^ | | 1.02 (0.99-1.06) | 0.05 | |
|  |  |  |  |  |  | Meta (MI)^#^ | 1.42 (1.22-1.66) | 9.00x10^-6^ | | 1.02 (0.99-1.06) | 0.14 | |
| rs147904962 | 17q21.31 | 45,377,171 | G/A | 0.11 | *ARHGAP27* | Discovery | 0.50 (0.37-0.67) | 4.73x10^-6^ | | 0.91 (0.85-0.98) | 9.29x10^-3^ | |
|  |  |  |  | 0.11 |  | Replication | 0.84 (0.56-1.27) | 0.42 | | 0.92 (0.83-1.03) | 0.16 | |
|  |  |  |  | 0.12 |  | Meta | 0.75 (0.66-0.85) | 4.08x10^-6^ | | 0.96 (0.93-0.99) | 3.97x10^-3^ | |
|  |  |  |  |  |  | Meta (MI)^#^ | 0.75 (0.66-0.85) | 4.15x10^-6^ | | 0.96 (0.93-0.99) | 4.17x10^-3^ | |

MAF: minor allele frequency, we listed MAF observed in the European subjects of the 1000 Genomes project, PanScan+PanC4 dataset, and PANDoRA samples, respectively; OR: odds ratio; CI: confidence interval.

* The meta-analysis for this SNP showed p < 0.05 in heterogeneity test

# Meta-analyses result after multiple imputation.

**Supplementary Table 3**. Associations of the correlated SNPs with PDAC risk under additive and recessive genetic models.

| **SNP** | **Proxy** | **R^2^** | **Recessive model** | | **Additive model** | |
| --- | --- | --- | --- | --- | --- | --- |
|  |  |  | **OR (95% CI)** | **P** | **OR (95% CI)** | **P** |
| rs2066357 |  |  | 0.78 (0.70-0.87) | 9.70x10^-6^ | 0.93 (0.88-0.97) | 1.89x10^-3^ |
| rs1339571 |  |  | 0.67 (0.56-0.80) | 7.25x10^-6^ | 0.93 (0.88-0.99) | 0.02 |
|  | rs61825243 | 0.991 | 0.67 (0.56-0.80) | 1.12x10^-5^ | 0.93 (0.88-0.99) | 0.02 |
| rs36018702 |  |  | 1.20 (1.11-1.30) | 4.85x10^-6^ | 1.07 (1.02-1.12) | 3.37x10^-3^ |
|  | rs6753785 | 0.9753 | 1.20 (1.11-1.30) | 5.39x10^-6^ | 1.07 (1.02-1.12) | 4.01x10^-3^ |
|  | rs4849442 | 0.9702 | 1.20 (1.11-1.30) | 5.50x10^-6^ | 1.07 (1.02-1.12) | 3.30x10^-3^ |
|  | rs3827536 | 0.928 | 1.17 (1.08-1.26) | 6.94x10^-5^ | 1.07 (1.02-1.11) | 6.56x10^-3^ |
|  | rs9308731 | 0.9184 | 1.17 (1.09-1.27) | 5.56x10^-5^ | 1.06 (1.02-1.11) | 9.21x10^-3^ |
|  | rs2241845 | 0.918 | 1.17 (1.08-1.26) | 6.80x10^-5^ | 1.06 (1.01-1.11) | 0.01 |
|  | rs6542334 | 0.9134 | 1.17 (1.08-1.26) | 5.97x10^-5^ | 1.06 (1.01-1.11) | 9.96x10^-3^ |
|  | rs1877331 | 0.9134 | 1.17 (1.08-1.26) | 6.34x10^-5^ | 1.06 (1.01-1.11) | 0.01 |
|  | rs2241843 | 0.9134 | 1.17 (1.08-1.27) | 6.10x10^-5^ | 1.06 (1.02-1.11) | 9.33x10^-3^ |
|  | rs4848393 | 0.9134 | 1.17 (1.08-1.26) | 8.48x10^-5^ | 1.06 (1.01-1.11) | 0.01 |
|  | rs13396983 | 0.9089 | 1.17 (1.08-1.26) | 5.91x10^-5^ | 1.06 (1.01-1.11) | 0.01 |
|  | rs6750599 | 0.9089 | 1.17 (1.08-1.26) | 7.64x10^-5^ | 1.06 (1.01-1.10) | 0.02 |
|  | rs7567444 | 0.9085 | 1.15 (1.07-1.25) | 3.06x10^-4^ | 1.06 (1.01-1.11) | 0.02 |
|  | rs2015454 | 0.9082 | 1.17 (1.08-1.26) | 9.74x10^-5^ | 1.06 (1.01-1.11) | 0.02 |
|  | rs6758181 | 0.8953 | 1.17 (1.09-1.27) | 5.48x10^-5^ | 1.06 (1.01-1.11) | 0.01 |
|  | rs6746608 | 0.8949 | 1.17 (1.08-1.26) | 7.37x10^-5^ | 1.06 (1.01-1.11) | 0.01 |
|  | rs17558117 | 0.8665 | 1.19 (1.10-1.29) | 3.98x10^-5^ | 1.06 (1.02-1.11) | 8.49x10^-3^ |
|  | rs616582 | 0.8665 | 1.19 (1.10-1.29) | 3.86x10^-5^ | 1.06 (1.02-1.11) | 8.01x10^-3^ |
|  | rs616130 | 0.8601 | 1.13 (1.05-1.22) | 1.47x10^-3^ | 1.05 (1.00-1.09) | 0.06 |
|  | rs6738028 | 0.8085 | 1.17 (1.07-1.28) | 3.60x10^-4^ | 1.06 (1.01-1.11) | 0.01 |
| rs4626538 |  |  | 0.84 (0.78-0.91) | 8.60x10^-6^ | 0.93 (0.89-0.97) | 1.61x10^-3^ |
|  | rs6947908 | 0.995 | 0.85 (0.78-0.91) | 1.91x10^-5^ | 0.93 (0.89-0.98) | 3.48x10^-3^ |
|  | rs6947939 | 0.995 | 0.85 (0.78-0.91) | 2.27x10^-5^ | 0.94 (0.89-0.98) | 3.65x10^-3^ |
|  | rs4421293 | 0.9852 | 0.85 (0.79-0.91) | 1.96x10^-5^ | 0.93 (0.89-0.97) | 2.06x10^-3^ |
|  | rs4335057 | 0.9852 | 0.85 (0.79-0.92) | 2.72x10^-5^ | 0.93 (0.89-0.98) | 3.31x10^-3^ |
|  | rs13231740 | 0.8576 | 1.04 (0.96-1.12) | 0.34 | 1.07 (1.02-1.12) | 3.53x10^-3^ |
|  | rs12538588 | 0.8527 | 1.04 (0.96-1.12) | 0.35 | 1.07 (1.02-1.12) | 3.67x10^-3^ |
|  | rs35019946 | 0.8436 | 1.04 (0.96-1.12) | 0.32 | 1.07 (1.02-1.12) | 3.26x10^-3^ |
| rs7008921 |  |  | 2.30 (1.59-3.32) | 8.85x10^-6^ | 1.06 (0.98-1.14) | 0.16 |
|  | rs17074078 | 0.9724 | 2.25 (1.56-3.25) | 1.66x10^-5^ | 1.04 (0.97-1.12) | 0.28 |
|  | rs17074056 | 0.9586 | 2.15 (1.49-3.10) | 4.29x10^-5^ | 1.04 (0.97-1.12) | 0.28 |
|  | rs7012234 | 0.8228 | 2.15 (1.41-3.28) | 3.75x10^-4^ | 1.05 (0.97-1.14) | 0.23 |
|  | rs7011163 | 0.8094 | 2.19 (1.44-3.34) | 2.58x10^-4^ | 1.06 (0.98-1.15) | 0.17 |
| rs147904962 |  |  | 0.50 (0.37-0.67) | 4.73x10^-6^ | 0.91 (0.85-0.98) | 9.29x10^-3^ |
|  | rs67622136 | 1 | 0.50 (0.37-0.67) | 6.02x10^-6^ | 0.91 (0.84-0.98) | 0.01 |
|  | rs79206700 | 0.9853 | 0.53 (0.39-0.71) | 2.95x10^-5^ | 0.92 (0.85-0.99) | 0.02 |
|  | rs56265946 | 0.9853 | 0.53 (0.39-0.71) | 3.02x10^-5^ | 0.92 (0.86-0.99) | 0.03 |
|  | rs12943205 | 0.9853 | 0.53 (0.39-0.71) | 2.91x10^-5^ | 0.92 (0.86-0.99) | 0.03 |
|  | rs34271337 | 0.9853 | 0.53 (0.40-0.72) | 3.96x10^-5^ | 0.92 (0.86-0.99) | 0.03 |
|  | rs12945838 | 0.9562 | 0.52 (0.39-0.71) | 2.31x10^-5^ | 0.92 (0.85-0.99) | 0.02 |
|  | rs140245332 | 0.9129 | 0.51 (0.37-0.70) | 2.49x10^-5^ | 0.92 (0.85-0.99) | 0.02 |
|  | rs36121378 | 0.9004 | 0.53 (0.39-0.72) | 4.11x10^-5^ | 0.92 (0.85-0.98) | 0.02 |
|  | rs36057737 | 0.9004 | 0.52 (0.38-0.70) | 2.47x10^-5^ | 0.91 (0.84-0.97) | 8.14x10^-3^ |
|  | rs16939964 | 0.9004 | 0.53 (0.39-0.72) | 4.62x10^-5^ | 0.91 (0.84-0.98) | 0.01 |
|  | rs12944705 | 0.9004 | 0.52 (0.39-0.71) | 2.93x10^-5^ | 0.92 (0.85-0.98) | 0.02 |
|  | rs867139 | 0.8703 | 0.57 (0.44-0.75) | 3.62x10^-5^ | 0.93 (0.86-0.99) | 0.03 |

**Supplementary Table 4.** Characteristics of genotyped samples from PANDoRA with additional Brazilian samples after quality control.

|  | **Cases** | **Controls** |
| --- | --- | --- |
| **Male, %** | 55.3 | 51.9 |
| **Median age, (25th-75th percentile)** | 65 (58-72) | 60 (50-68) |
| **Country, N** |  |  |
| Brazil | 70 | 250 |
| Czech Republic | 430 | 173 |
| Germany | 683 | 1018 |
| Greece | 109 | 16 |
| Hungary | 290 | 413 |
| Italy | 1298 | 1280 |
| Lithuania | 102 | 179 |
| Poland | 90 | 195 |
| Netherlands | 117 | 62 |
| United Kingdom | 93 | 134 |
| Total | 3282 | 3720 |

**Supplementary Table 5**. Associations of the 6 SNPs with PDAC risk under additive and recessive genetic models with additional Brazilian samples.

| **SNP** | **Locus** | **Position**  **(hg38)** | **Major/**  **minor** **allele** | **MAF** | **Closest gene** | **Phase** | **Recessive model** | | **Additive model** | | |  |
| --- | --- | --- | --- | --- | --- | --- | --- | --- | --- | --- | --- | --- |
|  |  |  |  |  |  |  | **OR (95% CI)** | **P** | | **OR (95% CI)** | **P** | |
| rs2066357 | 1p22.3 | 85,505,674 | G/A | 0.28 | *DDAH1* | Discovery | 0.78 (0.70-0.87) | 9.70x10^-6^ | | 0.93 (0.88-0.97) | 1.89x10^-3^ | |
|  |  |  |  |  |  | Replication | 1.25 (1.04-1.52) | 0.02 | | 1.14 (1.04-1.26) | 6.00x10^-3^ | |
|  |  |  |  |  |  | Meta* | 1.05 (0.76-1.43) | 0.78 | | 1.04 (0.89-1.22) | 0.60 | |
|  |  |  |  |  |  | Meta (MI)^#^ | 1.05 (0.77-1.43) | 0.79 | | 1.04 (0.89-1.21) | 0.61 | |
| rs1339571 | 1q25.3 | 184,598,799 | G/C | 0.18 | *C1orf21* | Discovery | 0.67 (0.56-0.80) | 7.25x10^-6^ | | 0.93 (0.88-0.99) | 0.02 | |
|  |  |  |  |  |  | Replication | 0.98 (0.74-1.31) | 0.90 | | 0.97 (0.88-1.06) | 0.48 | |
|  |  |  |  |  |  | Meta | 0.85 (0.79-0.91) | 1.26x10^-5^ | | 0.97 (0.95-0.99) | 0.02 | |
|  |  |  |  |  |  | Meta (MI)^#^ | 0.85 (0.79-0.92) | 1.96x10^-5^ | | 0.97 (0.95-1.00) | 0.02 | |
| rs36018702 | 2q13 | 111,170,796 | C/A | 0.45 | *BCL2L11* | Discovery | 1.20 (1.11-1.30) | 4.85x10^-6^ | | 1.07 (1.02-1.12) | 3.37x10^-3^ | |
|  |  |  |  |  |  | Replication | 0.98 (0.87-1.11) | 0.76 | | 0.99 (0.93-1.06) | 0.83 | |
|  |  |  |  |  |  | Meta | 1.08 (1.04-1.11) | 1.59x10^-5^ | | 1.03 (1.01-1.05) | 7.00x10^-3^ | |
|  |  |  |  |  |  | Meta (MI)^#^ | 1.07 (1.04-1.11) | 2.28x10^-5^ | | 1.03 (1.01-1.04) | 8.00x10^-3^ | |
| rs4626538 | 7q32.2 | 129,773,348 | T/G | 0.49 | *MIR96* | Discovery | 0.84 (0.78-0.91) | 8.60x10^-6^ | | 0.93 (0.89-0.97) | 1.61x10^-3^ | |
|  |  |  |  |  |  | Replication | 0.92 (0.81-1.04) | 0.18 | | 0.97 (0.91-1.03) | 0.30 | |
|  |  |  |  |  |  | Meta | 0.93 (0.90-0.96) | 3.44x10^-6^ | | 0.97 (0.95-0.99) | 9.00x10^-4^ | |
|  |  |  |  |  |  | Meta (MI)^#^ | 0.93 (0.90-0.96) | 5.20x10^-6^ | | 0.97 (0.95-0.99) | 1.00x10^-3^ | |
| rs7008921 | 8p23.2 | 5,566,886 | T/C | 0.09 | *RP5-991O23.1* | Discovery | 2.30 (1.59-3.32) | 8.85x10^-6^ | | 1.06 (0.98-1.14) | 0.16 | |
|  |  |  |  |  |  | Replication | 1.31 (0.71-2.43) | 0.39 | | 1.15 (0.84-1.56) | 0.39 | |
|  |  |  |  |  |  | Meta | 1.43 (1.22-1.67) | 6.29x10^-6^ | | 1.02 (0.99-1.06) | 0.14 | |
|  |  |  |  |  |  | Meta (MI)^#^ | 1.42 (1.22-1.66) | 8.49x10^-6^ | | 1.02 (0.99-1.06) | 0.14 | |
| rs147904962 | 17q21.31 | 45,377,171 | G/A | 0.11 | *ARHGAP27* | Discovery | 0.50 (0.37-0.67) | 4.73x10^-6^ | | 0.91 (0.85-0.98) | 9.29x10^-3^ | |
|  |  |  |  |  |  | Replication | 0.88 (0.58-1.33) | 0.54 | | 0.92 (0.82-1.03) | 0.14 | |
|  |  |  |  |  |  | Meta | 0.75 (0.66-0.85) | 5.40x10^-6^ | | 0.96 (0.93-0.99) | 4.00x10^-3^ | |
|  |  |  |  |  |  | Meta (MI)^#^ | 0.75 (0.66-0.84) | 3.15x10^-6^ | | 0.96 (0.93-0.99) | 3.00x10^-3^ | |

MAF: minor allele frequency observed in the European subjects of the 1000 Genomes project; OR: odds ratio; CI: confidence interval.

* The meta-analysis for this SNP showed P < 0.05 in heterogeneity test

# Meta-analyses results after multiple imputation.

**Supplementary Table 6.** Results of the gene-based analyses with MAGMA, comparing recessive model and additive model.

| **Gene** | **Chromosome** | **Start^a^** | **End^a^** | **Number of SNPs^b^** | **Number of parameters^c^** | **P_rec_^d^** | **P_add_^e^** |
| --- | --- | --- | --- | --- | --- | --- | --- |
| *CLPTM1L* | 5 | 1,315,752 | 1,347,099 | 44 | 3 | 7.06E-10 | 5.00E-10 |
| *TSGA13* | 7 | 130,666,643 | 130,689,432 | 38 | 8 | 2.66E-06 | 4.73E-09 |
| *KLF12* | 13 | 73,684,089 | 73,997,056 | 817 | 105 | 8.55E-06 | 2.46E-14 |
| *CFDP1* | 16 | 75,291,698 | 75,435,503 | 371 | 15 | 1.24E-04 | 6.22E-06 |
| *RHOV* | 15 | 40,870,214 | 40,876,234 | 18 | 3 | 2.44E-04 | 3.60E-04 |
| *CTSG* | 14 | 24,571,518 | 24,578,250 | 9 | 2 | **2.53E-04** | **0.20** |
| *CHAC1* | 15 | 40,950,962 | 40,958,512 | 13 | 3 | 3.29E-04 | 4.54E-04 |
| *BCAR1* | 16 | 75,226,181 | 75,270,053 | 174 | 15 | 3.31E-04 | 2.75E-09 |
| *LEPROTL1* | 8 | 30,093,408 | 30,179,208 | 104 | 15 | **4.34E-04** | **0.10** |
| *TMEM170A* | 16 | 75,441,054 | 75,467,497 | 83 | 3 | 4.67E-04 | 1.15E-03 |
| *CHST6* | 16 | 75,470,052 | 75,497,445 | 81 | 5 | 4.89E-04 | 3.62E-04 |
| *AMT* | 3 | 49,414,778 | 49,424,685 | 12 | 3 | 5.74E-04 | 2.72E-03 |
| *RNF34* | 12 | 121,398,041 | 121,432,623 | 92 | 10 | 6.29E-04 | 3.05E-05 |
| *GMPPB* | 3 | 49,714,844 | 49,725,951 | 11 | 3 | 9.59E-04 | 7.32E-04 |

^a^ Boundaries of each gene region (GRCh38).

^b^ Number of SNPs mapping to each gene region.

^c^ Number of principal components retained by MAGMA after pruning.

^d^ P-value of association between SNPs of each gene and PDAC risk, according to the recessive model.

^e^ P-value of association between SNPs of each gene and PDAC risk, according to the additive model.

Genes *CSTG* and *LEPROTL1* showed large differences between P_rec_ and P_add_ (in bold)
